# A primary hierarchically organized patient-derived model enables in depth interrogation of stemness driven by the coding and non-coding genome

**DOI:** 10.1038/s41375-022-01697-9

**Published:** 2022-09-21

**Authors:** Héléna Boutzen, Seyed Ali Madani Tonekaboni, Michelle Chan-Seng-Yue, Alex Murison, Naoya Takayama, Nathan Mbong, Elvin Wagenblast, Elias Orouji, Andrea Arruda, Amanda Mitchell, Faiyaz Notta, Mark D. Minden, Mathieu Lupien, Kerstin B. Kaufmann, John E. Dick

**Affiliations:** 1 Princess Margaret Cancer Centre, University Health Network, Toronto, ON M5G 0A3 Canada; 2 Department of Medical Biophysics, University of Toronto, Toronto, ON M5S 1A4 Canada; 3 PanCuRx Translational Research Initiative, Ontario Institute for Cancer Research, Toronto, ON M5G 0A3 Canada; 4 Department of Regenerative Medicine, Graduate School of Medicine, Chiba University, Chiba, Japan; 5 Princess Margaret Genomics Centre, University Health Network, Toronto, ON M5G 0A3 Canada; 6 Division of Medical Oncology and Hematology, Department of Medicine, University Health Network, Toronto, ON Canada; 7 Department of Medicine, University of Toronto, Toronto, ON Canada; 8 Ontario Institute for Cancer Research, Toronto, ON M5G 0A3 Canada; 9 Department of Molecular Genetics, University of Toronto, Toronto, ON M5S 1A8 Canada

**Keywords:** Cancer models, Cancer stem cells, Acute myeloid leukaemia

## Abstract

Many cancers are organized as cellular hierarchies sustained by cancer stem cells (CSC), whose eradication is crucial for achieving long-term remission. Difficulties to isolate and undertake in vitro and in vivo experimental studies of rare CSC under conditions that preserve their original properties currently constitute a bottleneck for identifying molecular mechanisms involving coding and non-coding genomic regions that govern stemness. We focussed on acute myeloid leukemia (AML) as a paradigm of the CSC model and developed a patient-derived system termed OCI-AML22 that recapitulates the cellular hierarchy driven by leukemia stem cells (LSC). Through classical flow sorting and functional analyses, we established that a single phenotypic population is highly enriched for LSC. The LSC fraction can be easily isolated and serially expanded in culture or in xenografts while faithfully recapitulating functional, transcriptional and epigenetic features of primary LSCs. A novel non-coding regulatory element was identified with a new computational approach using functionally validated primary AML LSC fractions and its role in LSC stemness validated through efficient CRISPR editing using methods optimized for OCI-AML22 LSC. Collectively, OCI-AML22 constitutes a valuable resource to uncover mechanisms governing CSC driven malignancies.

## Introduction

Acute myeloid leukemia is a heterogeneous disease [1–6] driven by leukemic stem cells (LSCs) [7–16]. Some AML patients achieve durable remission, but the majority relapse and die of their disease within 2 years. There is strong evidence that relapse arises from LSCs capable of surviving chemotherapy and initiating relapse [7–16]. A better understanding of mechanisms fueling stemness and chemoresistance in LSC is required to design efficient therapies. LSCs from across the spectrum of AML patients are heterogeneous in frequency, immunophenotype and genetic profile [15]. Despite this heterogeneity, all LSCs share the same functional stem cell hallmark that fosters long-term disease propagation: the capacity for self-renewal. The functional properties of LSC are directly linked to relapse [15] and poor prognosis [8, 9] establishing their clinical relevance. Numerous studies have established that the intrinsic stemness properties of LSCs from across diverse AML cohorts can be captured using gene expression signatures, many of which are shared with normal HSC [8, 9]. These LSC signatures are highly prognostic, demonstrating that stemness is capturing a shared property linked to clinical outcome within heterogeneous AML cohorts. The LSC17 score represents the most recent and validated proof of this concept. Indeed, this score was highly prognostic in multiple independent adult or pediatric AML datasets spanning >1000 AML samples [9, 17]. Thus regardless of the diverse paths taken during leukemogenesis, the high prognostic power of the LSC17 score suggests that many leukemogenic pathways converge onto their impact on stemness properties. The convergence of intrinsic stemness properties between AML patients has enabled the deployment of the LSC17 score into the clinic [18]. Collectively, these studies highlight the need to better understand the determinants that drive stemness in AML.

A number of key biological processes distinguish LSC from leukemia cells lacking stem cell activity. These include proteostatic responses, epigenetic pathways, immune escape ability and metabolism [5, 19–23] with a number translated into therapies [5, 6, 24–27]. However, difficulty in studying rare LSC populations represents a bottleneck for defining the regulatory processes that govern the stemness state. First, no markers exist to purify rare LSC populations to homogeneity [28]. This forces reliance on cumbersome, expensive and time-consuming xenograft assays for LSC detection for each patient sample [28, 29]. Second, each patient sample is limited in cell number and the rare LSC cannot be expanded since stemness is lost in culture [30]. Unfortunately, traditional human AML cell lines that have been grown for decades are of little use since they have lost many features of primary AML samples including a phenotypic and functional LSC-driven hierarchy [30]. Third, mechanistic interrogation of primary human LSC using standard genetic tools such as lentiviral transduction is possible but not efficient and non-lentiviral CRISPR editing of LSC has not yet been demonstrated. The reliance on lentivectors currently hinders functional validation of potential mechanisms driving stemness because it restricts identification of fundamental LSC features mostly to coding regions that represent only 2% of the genome [31]. There is increasing recognition that non-coding regulatory elements play important roles in controlling cell identities [16, 32]. Hence, it is crucial to identify human cellular models that recapitulate features of LSC-driven hierarchies characteristic of primary AML samples. Such models need to be easily genetically modified in ways that allow for interrogation of the entire coding and non-coding genomic landscape.

Here, we report the development of a patient-derived primary AML model, called OCI-AML22, that reflects the functional, transcriptional and epigenetic cellular hierarchy common to most primary AML samples, providing a powerful resource that permits investigation of the molecular basis of the stemness in LSC. OCI-AML22 can be efficiently modified with lentivectors and CRISPR-based methods, yielding a novel model that allows interrogation of stemness features that are critical for the future development of more efficient LSC-targeted therapies.

## Material and methods

Please, refer to Supplementary Material for detailed methods

## Results

### OCI-AML22 models the phenotypic and functional hierarchy of primary AMLs

**Fig. 1:**
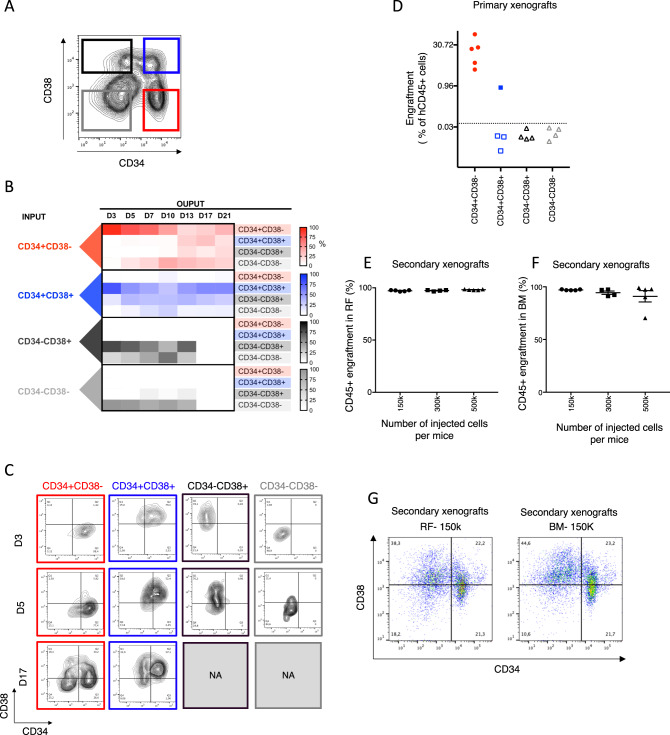
OCI-AML22 models the phenotypic and functional hierarchy found in primary AML. **A** Immunophenotypic profile of OCI-AML22 with the four fractions sorted as indicated based on CD34 and CD38 cell surface expression. **B** Immunophenotypic characterization of cellular outputs over time generated by each independently sorted and cultured fractions following in vitro expansion as assessed by flow cytometry. **C** Representative flow cytometric profiles of the independently sorted and cultured fractions over time; related to **B**. NA not available due to no viable cells remaining. **D** NSG mice were injected with sorted populations (100,000 cells per mouse) as indicated. Engraftment level was assessed 8 weeks after injection by flow cytometry measuring the percentage of human CD45+ (hCD45) cells in the injected femur. Each dot on the graph represents a mouse. **E**–**G** Cells collected from the xenografts generated after injection of OCI-AML22 fractions from **D** were pooled and sorted for human cells then injected into NSG-SGM3 mice at the indicated cell dose per mouse. Engraftment level (AnnexinV−, 7AAD−, hCD45+) was assessed 8 weeks later in the injected bone (RF). Each point represents a mice. **E** Non-injected bone, referred to as bone marrow (BM) (**F**). Representative FACS profiles of grafts in the right femur (left) or the bone marrow (right) are represented (**G**).

To establish an AML model that recapitulates a cellular hierarchy with LSCs at the apex, we screened AML patient samples (*n* = 34) for their ability to grow and expand in culture (Supplementary Fig. S1A and Table 1). Most AML samples could not be maintained for more than a few weeks and no sample consistently expanded in culture (Supplementary Fig. S1A), which is aligned with previous observations [30]. However, we identified one sample, from a relapse patient that could be expanded long term in vitro (Supplementary Fig. S1A, B) after optimization of culturing conditions. This cultured AML model is termed OCI-AML22. In vitro expansion resulted in an immunophenotypic hierarchy of four fractions defined by CD34 and CD38 cell surface expression (Fig. 1A and Supplementary Fig. S1B). The CD34+CD38− fraction was the only fraction able to maintain the entire phenotypic hierarchy in culture re-establishment assays (Fig. 1B, C). In parallel, functional xenotransplant LSC assays on each of the four CD34/CD38 subpopulations, sorted after 3–4 months of ex vivo expansion, established that only CD34+CD38− fractions contained LSC (Fig. 1D). All NSG mice (5/5) injected with 100,000 CD34+CD38− cells were engrafted at 8 weeks, while the same number of CD34+CD38+ or CD34− cells resulted either only in a single engrafted mouse (1 of 4 injected mice, 0.8% engraftment) or in no detectable engraftment, respectively (Fig. 1D). Robust engraftment following serial transplantation, that regenerated a full phenotypic hierarchy, conclusively established that CD34+CD38− LSC from primary mice had self-renewal potential [33] (Fig. 1E–G). The absolute LSC content within the CD34+CD38− fraction, quantified using limiting dilution xenograft assays, established that the LSC frequency was high (1/286; range: 1/102–1/804) (Table 2) when compared to the spectrum of LSC frequencies from fractions obtained from primary AML samples [9, 34] (Supplementary Fig. S2A). Collectively, these data establish that OCI-AML22 exhibits a phenotypic and functional hierarchy driven from LSC at the apex and has the capacity for both in vitro and in vivo long-term propagation.

**Table 1:** clinical characteristics of AML patient samples tested for in vitro expansion from Supplementary Fig. S1A.

Sample ID	Sex	Age at AML Dx	De novo vs. Secondary	Type of samples	Sample material	FAB	WBC at AML Dx	BM blast count at AML dx	MRC cytogenetics class at AML Dx	Initial Tx	NPM1 at dx	FLT3 ITD at Dx	FLT3 TKD at Dx
646	F	83.7	De novo	Diagnosis	PB	M5b	299	70	Intermediate (abnormal)	Supportive	nd	nd	nd
90240	F	54.2	De novo	Relapse3	Peritoneal fluid	M1	3	nd	Adverse	Induction	nd	nd	nd
80401	F	54	Secondary (MPN)	Diagnosis	PB	M1	24	60	Intermediate (abnormal)	Induction	nd	nd	nd
9682	F	76.6	De novo	Diagnosis	PB	Unclassified	34	nd	nd	Supportive	nd	nd	nd
80529	F	29.4	De novo	Diagnosis	PB	M4	21.1	60	Intermediate (normal)	Induction	Positive	Intermediate	Negative
90185	F	35.7	De novo	Diagnosis	PB	Unclassified	46	nd	Intermediate (normal)	Induction	Positive	Intermediate	Negative
80534	M	20.1	De novo	Relapse1—persistent	PB	M5a	54	95	Intermediate (normal)	Induction	nd	nd	nd
90736	F	49.9	De novo	Diagnosis	PB	Unclassified	41	nd	nd	Induction	nd	nd	nd
90707	M	59	De novo	Diagnosis	PB	M4	25	25	Intermediate (normal)	Induction	Positive	Negative	Negative
90784	F	61.9	De novo	Diagnosis	PB	M5a	59	90	Intermediate (normal)	Positive	High	Negative	
858	M	55.9	Secondary (MPN)	Diagnosis	PB	M1	33	70	Intermediate (normal)	Induction	Positive	Negative	Negative
100052	M	79.2	Secondary (MDS)	Diagnosis	PB	M4	38	60	Adverse	Supportive	nd	nd	nd
100016	M	73.2	De novo	Diagnosis	PB	M5a	20	80	Intermediate (normal)	Induction	Negative (K)	Negative (K)	Negative (K)
100006	F	63.3	De novo	Diagnosis	PB	M5a	25	80	Intermediate (abnormal)	Induction	nd	nd	nd
100112	M	69.5	Secondary (MDS)	Persistent disease	PB	nd	16	80	Intermediate (normal)	Induction	Negative	Negative	Negative
100116	F	65.3	De novo	Diagnosis	PB	M4	46	nd	nd	Supportive	nd	nd	nd
100118	M	61.8	De novo	Persistent disease	PB	M1	204	90	Intermediate (normal)	Induction	Negative	Intermediate	Negative
100127	F	29.2	De novo	Relapse1	PB	Unclassified	10	nd	nd	Induction	nd	nd	nd
100183	M	69.8	De novo	Diagnosis	PB	M5a	88	85	Intermediate (normal)	Induction	Positive	Negative	Negative
100207	M	71.9	De novo	Diagnosis	PB	M3	62	nd	Favorable	Induction	nd	nd	nd
100249	F	67.1	De novo	Diagnosis	PB	Unclassified	282	95	nd	Induction	Positive	Intermediate	Negative
100255	M	59.6	De novo	Relapse1	PB	M4	108	50	Intermediate (normal)	Induction	Positive	Negative	Positive
80014	M	52	Secondary (rads)	Diagnosis	PB	M4Eo	35.9	90	Favorable	Induction	nd	nd	nd
90520	M	61.7	De novo	Diagnosis	PB	Unclassified	103	39	Intermediate (normal)	Induction	Positive	Negative	Positive
100274	M	59.6	De novo	Relapse1	PB	M4	108	50	Intermediate (normal)	Induction	Positive	Negative	Positive
100286	M	50.5	De novo	Diagnosis	PB	M1	77.2	90	Intermediate (abnormal)	Induction	nd	nd	nd
90272	F	29.2	De novo	Diagnosis	PB	Unclassified	10	nd	nd	Induction	nd	nd	nd
90517	M	61.8	De novo	Persistent disease	PB	M1	204	90	Intermediate (normal)	Induction	Negative	Intermediate	Negative
90047	F	58.3	De novo	Diagnosis	PB	M4	46.9	40	Intermediate (normal)	Induction	Positive	Negative	Negative
100347	F	63.3	De novo	Diagnosis	PB	M1	89.9	90	Intermediate (normal)	Induction	Negative	Negative	Negative
100454	M	62.8	Secondary (MPN)	Diagnosis	PB	Unclassified	27	50	Adverse	Supportive	nd	nd	nd

**Table 2:** Limiting dilution assay at 12 weeks.

Fraction	Dose	Tested	Response	Lower frequency	Estimated frequency	Upper frequency
CD34+CD38−	30,000	3	3	1/803.9	1/286.4	1/102
	7500	4	4			
	1875	6	6			
	469	5	4			

OCI-AML-22 cells were expanded in culture for 4 months before CD34+CD38− cells were isolated and injected into NSG-SGM3 mice at the indicated cell doses. The number of injected mice (tested) and engrafted (response) as well as lower, estimated and upper LSC frequencies are indicated 12 weeks after injection.

### OCI-AML22 maintains the polyclonal genetic architecture of the primary AML sample

**Fig. 2:**
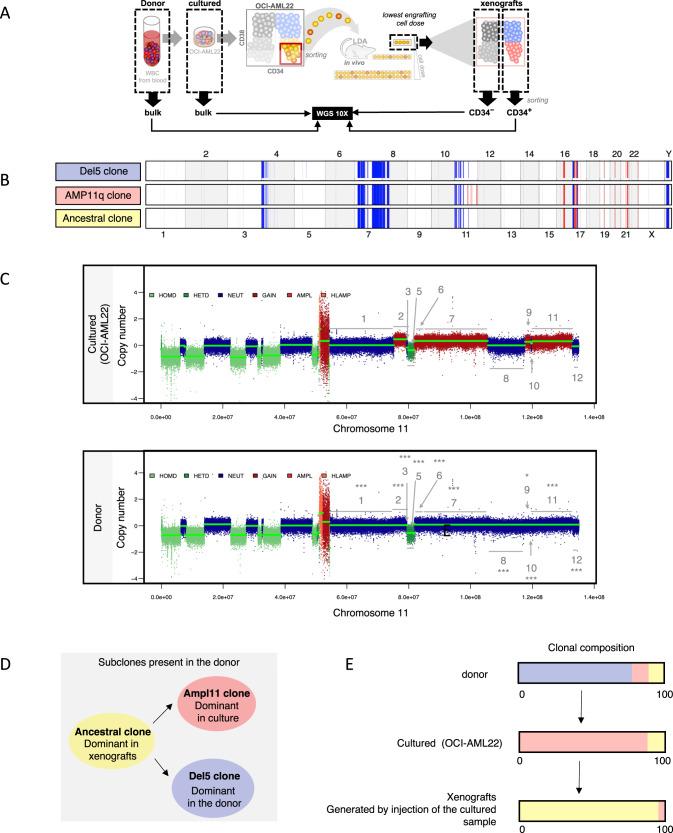
OCI-AML22 maintains the polyclonal genetic architecture of the primary AML sample. **A** Schematic representation of the samples sequenced for WGS. **B** Alterations present in each of the dominant clones are displayed. Copy number losses are defined as CN less than 1.5 (shown in blue) while gains have CN greater than 2.5 (shown in red). The width of the colored region corresponds to the size of the modified region in the genome. **C** Representation of the copy number obtained on chromosome 11 for cultured and donor samples. Segment regions that were detected using hmmcopy in the cultured sample (top) were used to break down the chr11 arm q of the donor sample (bottom) into matching regions. Within these segments, each dot (at 1 kb intervals) was taken to compare the average copy number to the adjacent segments and thus determine if the average copy numbers were different between the indicated adjacent regions. Wilcox tests were run. Each start on top of each region shows the existence of amplifications that can be significantly detected at a subclonal level in the donor sample. **D** Evolutionary relationships of subclones present in the donor sample. The sample where the clone is dominant is indicated. **E** Clonal composition of the different sequenced samples using the same clones color coding as in **B** and **D**.

Since the donor sample from which OCI-AML22 was derived had complex cytogenetics, we assessed its genetic stability during in vitro and in vivo expansion. Whole genome sequencing (WGS) was undertaken on the primary donor sample (day 0), the cultured OCI-AML22 model (~day 30), and on xenografts generated from CD34+CD38− cells sorted from the cultured sample (~day 120) (Fig. 2A). Genomic analysis showed that cultured and donor samples were highly similar with 99% conservation. The CD34+ fraction from xenografts showed an even higher conservation (99.8%). Detailed analysis further revealed that the genetic differences between the dominant clones of the cultured, xenografted and donor samples were reflective of subclonal genetic diversity, where the dominant populations of the culture and xenografts arose from rare preexisting subclones present in the donor sample (Fig. 2B–D and Supplementary Fig. S3A and Tables 3 and 4). WGS showed that the dominant clone in the OCI-AML22 cultured sample exhibits a series of amplifications on chromosome 11 (Supplementary Fig. S3A, arrow 1). This clone was termed clone AMP11 (Supplementary Fig. S3A, line 2 and Fig. 2B, line 2, in red). This series of amplifications on chromosome 11 is not present in the dominant clone of the donor sample (Supplementary Fig. S3, arrow 1, line 1). However, a focused copy number analysis of this region on chromosome 11 in the donor sample showed that these alterations were already present at a subclonal level (Fig. 2C and Tables 3 and 4). Thus the series of 11q amplifications were not generated by a genetic drift due to culture, but arose by the selective amplification of a minor clone preexisting in the donor sample. Of note, the AMP11 clone could also be detected at the subclonal level in xenografts generated by the cultured sample (Tables 3 and 4). WGS also revealed that the dominant clone in the donor (Supplementary Fig. S3A, arrow 1) contains a small deletion on chromosome 5 (and lacking the 11q amplification) (Supplementary Fig. S3A, arrow 2); termed clone del5 (Supplementary Fig. S3A, line1 and Fig. 2B, line 1, in blue). By contrast, the dominant clone in xenografts, generated by the cultured sample, lacked both the chromosome 5 deletion and the chromosome 11q amplifications (Supplementary Fig. S3A, line 3). Since a deletion cannot be spontaneously restored to normal, the clone that preferentially expands in vivo must be ancestral to both the del 5 and the AMP11 clones. This clone is termed the ancestral clone (Supplementary Fig. S3A, line 3 and Fig. 2B, line 3 in yellow). Thus, in vitro culture, followed by in vivo propagation of the donor sample, provided insight into the evolutionary relationships of the three genetic subclones present at different levels in the original donor sample (Fig. 2D). These include the del5 clone, dominant in the donor sample (Fig. 2B, D, E, in blue), the minor AMP11 clones, and the ancestral clone able to generate both del5 and AMP11 subclones (Fig. 2B, D, E, in yellow). The ancestral clone must be present in both the donor and in culture at subclonal levels since it becomes detectable following xenotransplantation of the cultured OCI-AML22 cells (Fig. 2E, in yellow). The AMP11 clone, present at the subclonal level in the donor, preferentially expands in culture and remains present but at the subclonal levels in xenografts generated by the cultured sample (Fig. 2B, D, E, in red). Altogether, the WGS analysis revealed that the cultured OCI-AML22 model is genetically stable during prolonged in vitro and in vivo propagation, while maintaining at least two of the clones originally present in the donor sample: the ancestral clone and the AMP11q clone. These results are reminiscent of our findings documenting leukemia evolution using xenografts [15, 35, 36] and highlight that the OCI-AML22 model maintains the polyclonal genetic architecture characteristic of primary AML samples in contrast with traditional AML cell lines that are clonal.

**Table 3:** Chromosome 11q fragments being compared coordinates.

Fragments being compared	Coordinates
1	chr11:54526001-75256001
2	chr11:75256001-79404001
3	chr11:79404001-79816001
4	chr11:79816001-81783001
5	chr11:81783001-81826001
6	chr11:81826001-81900001
7	chr11:81900001-105587001
8	chr11:105587001-117653001
9	chr11:117653001-119635001
10	chr11:119635001-119932001
11	chr11:119932001-132956001
12	chr11:132956001-135087001

Segment regions that were detected using hmmcopy in the cultured sample and were used to break down the chr11 arm q of the donor sample or the fractions sorted from xenografts.

**Table 4:** Comparisons of fragments between the donor or xenografts fractions (CD34+ or CD34−) and the cultured sample.

	In the donor sample		CD34 positive from xenografts		CD34 minus from xenograft		
Comparison	Wilcox.*p* value	Significance	Wilcox.*p* value	Significance	Wilcox.*p* value	Significance	In either 34p or cd34m
1 to 2	1.11E-267	***	0.523362	ns	0.087603	ns	ns
2 to 3	3.80E-218	***	3.93E-239	***	1.47E-240	***	***
3 to 4	7.64E-06	***	0.461146	ns	0.714475	ns	ns
4 to 5	2.01E-25	***	3.27E-28	***	3.92E-27	***	***
5 to 6	1.88E-17	***	1.56E-18	***	1.03E-17	***	***
6 to 7	4.44E-46	***	1.58E-48	***	2.78E-48	***	***
7 to 8	0	***	0.722962	ns	0.004922	***	***
8 to 9	3.96E-09	***	0.000187	***	9.39E-16	***	***
9 to 10	0.0387516	*	2.05E-06	***	5.40E-05	***	***
10 to 11	6.89E-12	***	0.000777	***	0.25894	ns	***
11 to 12	4.85E-125	***	0.000819	***	5.27E-14	***	***

Segment regions that were detected using hmmcopy (Table 3) in the cultured sample were used to break down the chr11 arm q of the donor sample, or xenografts into matching regions. Within these segments, each dot (at 1kb intervals) was taken to compare the average copy number to the adjacent segments and thus determine if the average copy numbers were different between the indicated adjacent regions. Wilcox tests were run. Significance shows the existence of amplifications that can be detected at a subclonal level in the interrogated sample: donor sample or xenografts (CD34+ fraction, CD34− fraction or both). *p* < 0.05: *, *p* < 0.01:**, *p* < 0.001: ***.

### OCI-AML22 preserves a transcriptional and epigenetic landscape of primary stem cells throughout ex vivo expansion

**Fig. 3:**
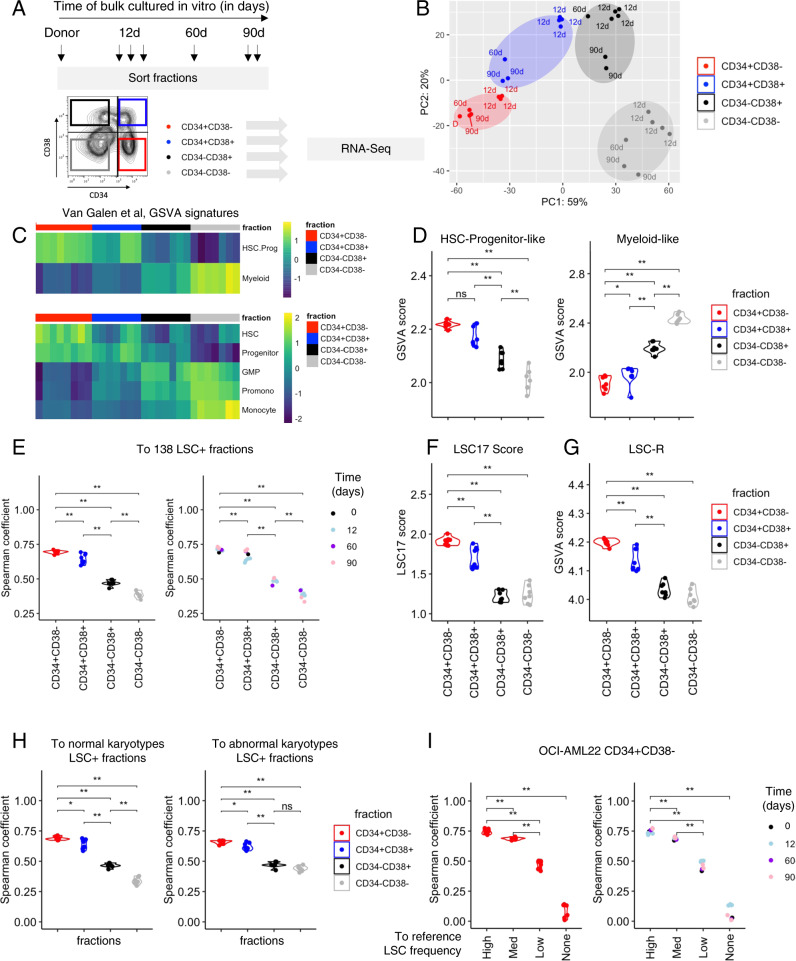
The OCI-AML22 CD34+CD38− fraction preserves the transcriptional and epigenetic landscape of primary stem cells throughout ex vivo expansion. **A** Schematic representation of OCI-AML22 sorting strategy applied for RNA-Seq. Each arrow indicates an independently expanded culture. These fractions are used throughout the RNA-Seq analysis. **B** Principal component analysis (PCA) of RNA-seq data generated from CD34+CD38− (red), CD34+CD38+ (blue), CD34−CD38+ (black) and CD34−CD38− (gray) subpopulations. **C** Supervised heatmap clustering for GSVA scores calculated for the van Galen signatures [10] and organized based on OCI-AML22 sorted fraction. Pathways indicative of primitive-like AML cells (HSC-like, Progenitor-like are combined into the HSC-Prog-like signature) while the others (GMP, Promo and Monocyte) are combined in the Myeloid signature. **D** GSVA score of genes present in the HSC-Progenitor-like (left), Myeloid-like (right) signatures (Van galen et al.) across OCI-AML22 fractions. **E** Spearman correlation calculated for the gene expression of the LSC104 genes [9] of each OCI-AML22 fractions compared to the average gene expression of these genes across 138 primary AML fractions, enriched for functional LSC described in ref. [9]. Fractions are colored depending of the fraction type (left) or the time OCI-AML22 has been maintained in culture before the sort (right). **F** LSC17 score calculated for each OCI-AML22 fraction. **G** Gene set variation analysis (GSVA) score of genes present in the LSC-R signature [8] across OCI-AML22 fractions. **H** Spearman correlation coefficient calculated between the LSC104 signature of each of the OCI-AML22 indicated fraction at the bottom, and the LSC104 signature from a group of normal karyotype LSC+ (60 fractions) (left) or a group of abnormal karyotype LSC+ fractions (55 fractions) (right). **I** Spearman correlation coefficient comparing the LSC104 signature of the OCI-AML22 CD34+CD38− fractions, to each of the LSC104 signatures for groups of samples indicated on the bottom (LSC frequency High, Medium (Med), low or no detectable LSC activity (LSC neg). Points are colored according to the OCI-AML22 fraction sorted (left) or depending on the time OCI-AML22 has been kept in culture before sorting the CD34+CD38− fraction; 12d : 12 days, 60d : 60 days, 90d : 90 days, D: primary donor)(right).

To determine if the transcriptional signature of the CD34+CD38− fraction was maintained over time, we performed deep RNA sequencing (RNA-seq) of CD34+CD38− cells from the donor sample and four immunophenotypic fractions harvested at multiple time points of culture (12, 60, 90 days) (Fig. 3A). Principal component analysis (PCA) showed that even after 2 to 3 months of in vitro culture, the CD34+CD38− fractions of OCI-AML22 and the donor sample clustered together indicating that ex vivo expanded cells preserved the global transcriptomic landscape of its original donor (Fig. 3B). In accordance with our functional data (Fig. 1), CD34+CD38+ populations were positioned in the PCA space between the CD34+CD38− engrafting and non-engrafting CD34− fractions. CD34− fractions clustered together and were the most distinct from CD34+CD38− populations. The functional hierarchical organization described in Fig. 1 prompted us to investigate whether OCI-AML22 recapitulates LSC and non-LSC features extracted from heterogenous primary AML cohorts, as well as the diverse AML cellular states recently extracted from scRNA-seq of primary AML samples [10]. Gene set variation analysis (GSVA) on each of the OCI-AML22 fractions showed that the OCI-AML22 model recapitulates the various cellular states extracted from primary AML samples with enrichment of primitive states in the functional LSC fractions (HSC, Progenitor, summarized as HSC.Prog) and progressive enrichment for mature states in the non-LSC containing fractions (GMP, Pro-mono, Monocyte, summarized as Myeloid) (Fig. 3C, D and Supplementary Fig. S4A–E). Similarly, using chromatin accessibility signatures generated from highly purified fractions obtained from the normal hematopoietic system [32], we show that the OCI-AML22 CD34+ fraction was enriched in stem cell signatures (LT/HSPC and Act/HSPC), while the CD34− non-engrafting fraction was enriched for signatures of mature myeloid populations (granulocyte, monocyte signatures) (Supplementary Fig. S4F). All OCI-AML22 fractions showed the lowest concordance with the erythroid and lymphocyte (T cells and B cells) signatures as compared to mature myeloid populations (granulocyte, monocyte). This result is concordant with a block of differentiation and a shift toward myeloid pathways characteristic for AML (Supplementary Fig. S4F).

To determine whether OCI-AML22 CD34+CD38− fraction recapitulates the stem cell transcriptional programs shared with primary LSCs, we used the LSC104 signature, a stem cell tool whose clinical relevance has been demonstrated in multiple cohorts [9]. The stemness properties of any group of samples can be determined on the basis of a spearman correlation coefficient to the average gene expression of each of these 104 LSC+ genes [9]. First we compared the gene expression values of the reference set of 104 LSC-specific genes generated on a group of 138 primary LSC+ fractions (LSC+ reference), to LSC104 gene expression values calculated for each of the OCI-AML22 fractions obtained at different time points. The resulting correlation score was the highest when comparing the OCI-AML22 CD34+CD38− fractions to the LSC+ reference; the score gradually decreased alongside the reduced engraftment ability of the fractions with the lowest coming from the non-engrafting CD34−CD38− fractions (Fig. 3E, left). These results remained consistent, independent of the time the OCI-AML22 model was maintained in culture (Fig. 3E, right). The same conclusions were validated using three independent approaches: LSC17 scoring [9] across all fractions (Fig. 3F); GSVA scoring using the LSC-R signature [8], a previous stemness signature (Fig. 3G); and GSVA scoring using the HSC-R signature, a stemness signature generated from normal hematopoietic stem cells [8] (Supplementary Fig. S4G). This demonstrates that the OCI-AML22 hierarchy recapitulates the stemness features of a diverse cohort of primary samples.

Although the OCI-AML22 model is derived from a complex cytogenetic sample, we validated that LSC from OCI-AML22 are representative of stemness properties exhibited by primary AML samples regardless of karyotype. Indeed, correlation of the LSC104 gene expression values generated from the CD34+CD38− OCI-AML22 fraction was similar when compared to the LSC104 average values generated from either LSC+ fractions of normal karyotype (*n* = 60) or abnormal karyotype (*n* = 55) samples (Fig. 3H). Of note, both normal and abnormal karyotype spanned similar LSC frequency ranges (Supplementary Fig. S5A). Finally, since the LSC+ fractions obtained from primary AML samples present a broad range of LSC frequencies, we generated LSC104 correlation data of four groups of primary AML fractions, based on their LSC frequency: none, low, medium, and high, to determine which group is the most reflective of the OCI-AML22 LSC population. The LSC104 gene expression values of the the OCI-AML22 CD34+CD38− fraction correlated best with the LSC104 values of the highest LSC frequency group (Fig. 3I, left), and this was independent of the culture time (Fig. 3I, right), confirming the strong LSC enrichment in the OCI-AML22 LSC fraction. Altogether, our data establishes that the OCI-AML22 LSC fraction captures the transcriptional and epigenetic stemness programs of highly purified LSC fractions obtained from patients across a wide spectrum of clinical and genetic properties (Supplementary Fig. S6) and these are preserved during culture.

### OCI-AML22 enables functional interrogation of leukemia stemness properties

**Fig. 4:**
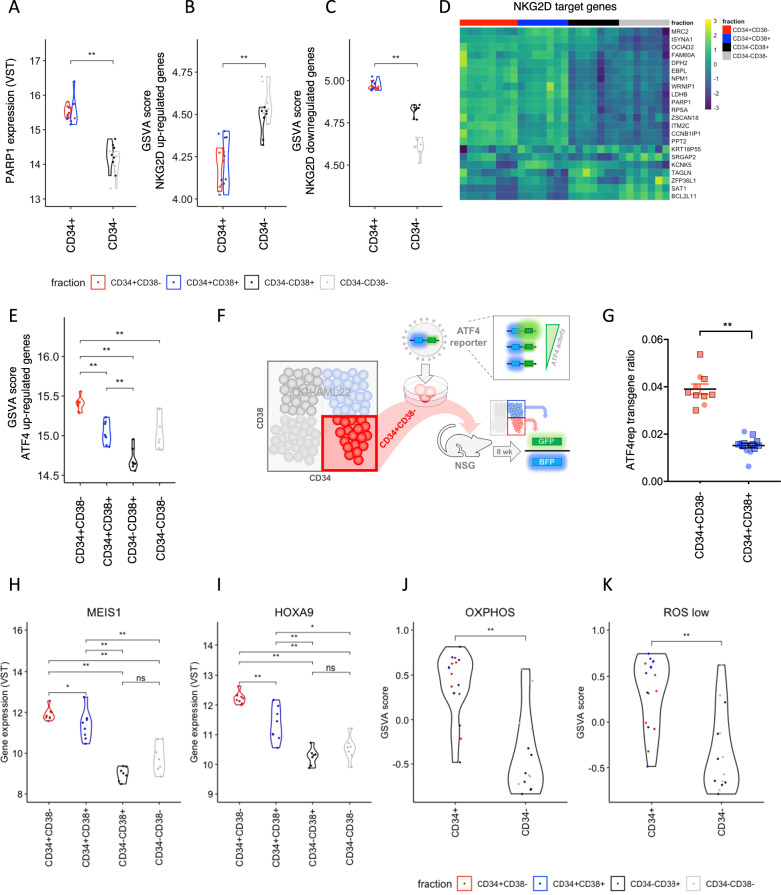
OCI-AML22 enables functional interrogation of leukemia stemness properties. **A** PARP1 expression of OCI-AML22 fractions was determined by RNA-Seq. GSVA score for NKG2D upregulated (**B**) or downregulated (**C**) genes as described in ref. [21] across OCI-AML22 sorted fractions as indicated in Fig. 2A. **D** Supervised heatmap clustering of NKG2D target gene expressions of sorted OCI-AML22 fractions. **E** GSVA score of ATF4 upregulated genes as described in ref. [20] across individual OCI-AML22 fractions as extracted in Fig. 2B. **F** Experimental scheme. **G** OCI-AML22 CD34+CD38− cells were transduced with ATF4 lentiviral fluorescent reporter (ATF4rep) to track ATF4 transcriptional activity. BFP and GFP were assessed on viable human CD45+ cells extracted from the injected femora of NSG mice injected with 100k (round) or 200k (square) transduced cells, 8 weeks post xenotransplantation. The ATF4rep transgene ratio (GFP/BFP; BFP as internal transduction control) was then calculated for each indicated fractions of the 7AAD-AnnexinV− hCD45+ population from xenografts. Error bars (s.e.m) are indicated. The expression of MEIS1 (**H**) and HOXA9 (**I**) was assessed across OCI-AML22 sorted fractions as indicated in Fig. 2A. **J** GSVA score for genes that are part of the OXPHOS High signature [41], across OCI-AML22 sorted fractions as indicated in Fig. 2A. **K** GSVA score for genes that are part of the ROSLow signature [42], across OCI-AML22 sorted fractions as indicated in Fig. 2A.

To determine if the OCI-AML22 model can be used to interrogate molecular determinants driving stemness, we tested a variety of biological properties that were previously reported to discriminate populations within the cellular hierarchy of primary AML samples. We first tested the potential of the OCI-AML22 model to give insights into LSC-specific immune evasion properties since recent studies of 177 primary AML samples showed that LSC, but not their non-stem cell progeny, are able to evade Natural Killer (NK)-driven anti-tumor immunity. LSCs upregulate PARP1 whose overexpression downregulates the activity of NKG2D ligands that are recognized by NK cells [21]. As expected, PARP1 expression was higher in engrafting (CD34+) vs. non-engrafting (CD34−) OCI-AML22 fractions (Fig. 4A). GSVA showed that genes known to be upregulated by NKG2D ligands were less enriched in CD34+ compared to CD34− fractions (Fig. 4B). Inversely, genes downregulated by NKG2D ligands were more enriched in CD34+ compared to CD34− fractions (Fig. 4C, D). Second, GSVA on each OCI-AML22 fraction showed that ATF4 upregulated genes were significantly enriched in CD34+CD38− fractions as compared to other non-engrafting fractions (Fig. 4E and Supplementary Fig. S7A). This was also validated in vivo, using the ATF4 biosensor (Fig. 4F, G and Supplementary Fig. S7B–E). These data confirm that the integrated stress response (ISR) is regulated across the OCI-AML22 hierarchy in the same way as previously described across primary normal or LSC hierarchies, where the ISR is closely associated with LSC function [20, 37]. Moreover, HOXA9 and MEIS1 are key transcription factors well known to be overexpressed in LSC compared to non-LSC [11] and to be correlated to poor prognosis [38], which we have confirmed across the OCI-AML22 hierarchy (Fig. 4H, I). Additionally, many studies have shown that LSCs harbor a characteristic energy metabolism centering on a high oxidative phosphorylation signature [39–41]. We confirmed that this pathway is also upregulated in the LSC+ OCIAML22 fractions compared to the non-LSC fractions (Fig. 4J). Finally, we confirmed that the ROSLow signature [42] that discriminates LSC+ from LSC− fractions in primary AML samples also discriminates LSC+ from LSC− fractions in OCI-AML22 (Fig. 4K).

Collectively, these data validate that the OCI-AML22 hierarchical structure recapitulates key hallmarks previously reported for primary AML samples, both in vitro and in vivo. Thus, OCI-AML22 represents a good model to study LSCs and interrogate stemness properties.

### Clusters of cis-regulatory elements (CORE) discriminate LSC from non-LSC fractions in primary AML samples

**Fig. 5:**
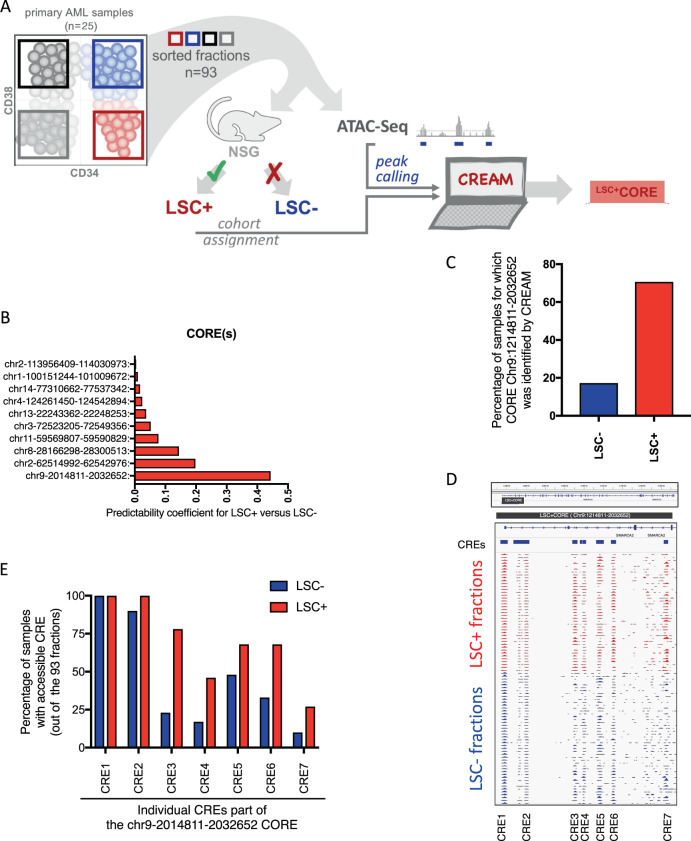
Chromatin accessibility profile of LSC enriched and depleted fractions identifies COREs discriminating LSC+ from non-LSC fractions. **A** Schematic representation of the experimental strategy, **B** coordinates and predictability coefficient for LSC+ vs. LSC− for CORE that are detected in either LSC+ or LSC− fractions. **C** Percentage of LSC− and LSC+ fraction for which CREAM algorithm detects the CORE-chr9 2014811-2032652, out of the 93 fractions sequenced, **D** IGV representation of the CORE-chr9 2014811-2032652 for primary AML LSC+ and LSC− fractions, **E** Percentage of LSC+ or LSC− primary AML fractions for which CRE part of the CORE of interest are accessible out of the 93 fractions sequenced.

We next set out to uncover potential new stemness regulators associated with LSC in primary AML, as a basis to demonstrate the power of OCI-AML22 to provide new insights into LSC biology. Chromatin accessibility of non-coding genomic regions has greater power in predicting cell state identity as compared to gene expression [16]. We established a new discovery approach to identify functionally relevant non-coding regions that define the LSC state as compared to non-LSC populations. In the human genome, 20–40% of the non-coding regions are predicted to be covered by cis-regulatory elements (CRE) [38]. These elements are not evenly spread across the human genome and can be either isolated or clustered together, suggesting that coordinated, fine-tuned regulation occurs at these clusters and they are acting as larger entities of biological relevance. Clusters of cis-regulatory elements (COREs) are regions of high CRE density defined by chromatin accessibility which we identified using the machine learning algorithm CREAM [39]. They have been shown to reliably discriminate cell identities across multiple cancer cell types. Hence, we hypothesized that CREAM could identify COREs able to distinguish LSC from their non-LSC counterparts thereby uncovering new regulators of stemness governing LSC biology. CREAM was applied to chromatin accessibility data generated on LSC containing (LSC+; *n* = 41) and LSC depleted (LSC−; *n* = 52) fractions that were isolated from 25 uncultured AML patient samples and functionally validated as described previously [9] (Fig. 5A). According to the calculated predictability coefficient, the CORE with the highest potential to distinguish LSC+ from LSC− fractions was located within intronic regions of chromosome 9 (CORE-chr9-2014811-2032652) (Fig. 5B) and was detected in the majority (>75%) of the LSC+ fractions but only in 20% of the LSC− fractions (Fig. 5C). CREAM identified this CORE as encompassing seven individual CREs located within intronic regions of the SMARCA2 locus (Fig. 5D), and that were accessible in different proportions of LSC+ compared to LSC− fractions (Fig. 5E). Overall, the recurrent pattern defined by CORE-chr9-2014811-2032652 (LSC + CORE) across LSC+ vs. LSC− suggests that individual CREs of this CORE might be particularly relevant for LSC function, but functional validation is required.

### Functional interrogation of individual CRE located within CORE-chr9-2014811-2032652 through OCI-AML22 LSC CRISPR/Cas9 editing

**Fig. 6:**
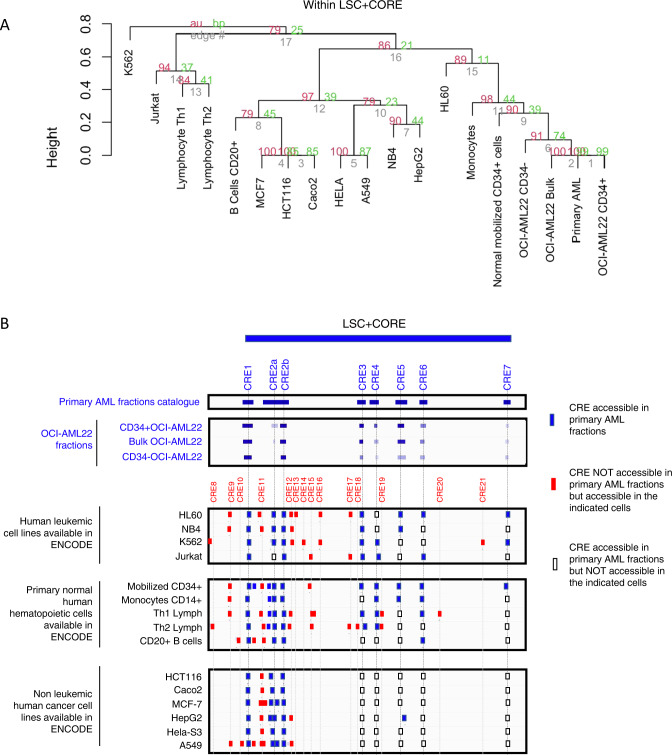
OCIAML22 recapitulates chromatin accessibility exhibited by primary AML samples within the CORE-chr9 2014811-2032652 locus. **A** Hierarchical clustering using CRE elements within the CORE of interest (CORE-chr9-2014811_2032652) across the indicated samples. **B** Chromatin accessibility profiles within the CORE of interest, for the indicated samples. CRE accessible in primary AML fractions have been highlighted in dark blue, CRE not accessible in primary AML fractions but present in the indicated cells have been highlighted in red, CRE accessible in primary AML fractions but not accessible in the indicated cells are surrounded with a black line as indicated in the legend.

Functional studies require an experimental model, so we first mined ENCODE. No cell lines showed a similar pattern of accessibility peaks as found in our set of 93 primary AML sorted fractions (Fig. 6A, B). By contrast, there was a highly concordant accessibility pattern of the LSC+ CORE region between OCI-AML22 fractions and the catalog of peaks obtained from 93 primary AML fractions (Fig. 6A, B). These results demonstrate the superiority of OCI-AML22, compared to any other AML cell lines, at recapitulating chromatin accessibility features characteristic of LSCs.

**Fig. 7:**
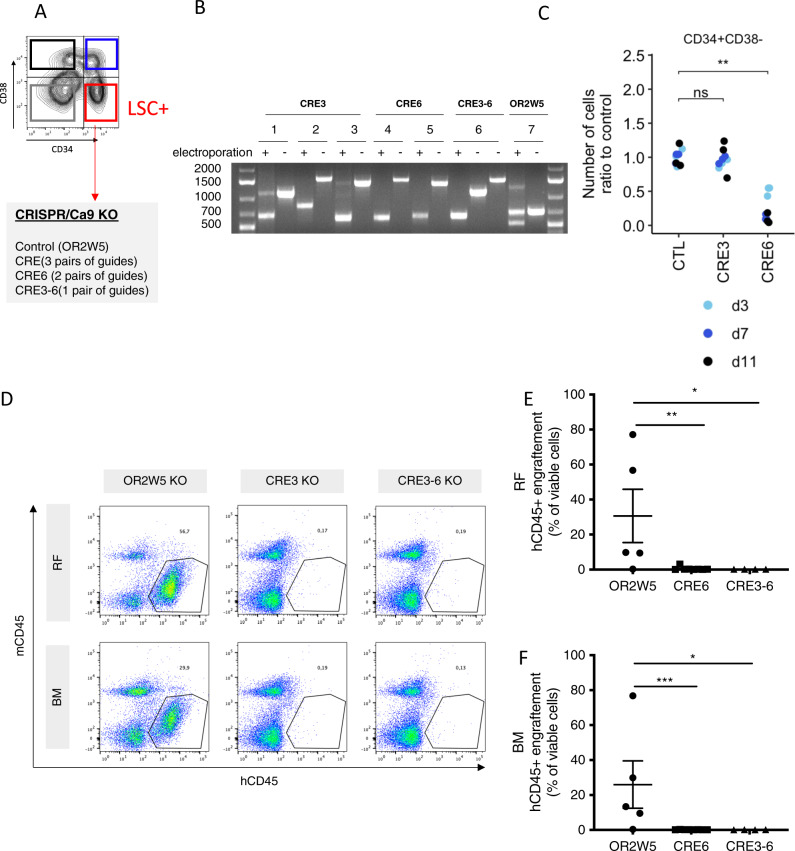
CRISPR/Cas9 in OCIAML22 LSC fraction allows to functionally interrogate individual CRE located within the CORE of interest. **A** Schematic representation of the CRISPR editing strategy. **B** Fragments generated after PCR of the CRISPR edited (electroporated) or non CRISPR edited (not electroporated) after KO of the indicated elements: CRE3, CRE6 or the entire region from CRE3 to CRE6 (CRE3-6) or control OR2W5, using PCR guides that are flanking the region to KO. The shorter band represents the fraction of KO cells while the longer band represents the proportion of cells not KO. **C** Total cells expanded in culture at the indicated time points after CRISPR editing of the regions of interest in the OCIAML22 LSC fraction. **D**–**F** The OCIAML22 LSC fraction was CRISPR edited then cells were expanded for 22 days before injection in NSG mice. After 21 weeks, the engraftment was assessed. representative FACS profile (**D**) and engraftment level in either the injected femur (right femur, RF) (**E**) or Bone marrow (BM) (**F**) are represented. Error bars (s.e.m) are indicated.

To functionally test if LSCs depend on individual CREs found within the LSC+ CORE locus, we specifically targeted 2 CREs in the LSC fractions of OCI-AML22 using CRISPR/Cas9 methods we optimized. The OCI-AML-22 LSC fraction was used to either individually knock-out (KO) CRE3 or CRE6 or delete the entire flanking region (Fig. 7A, B and Table 5). Effective KO of each of the regions of interest was confirmed by PCR/gel electrophoresis and drop out of either CRE3 or CRE6 was observed for any of the gRNA pairs selected (Fig. 7B). Of note, control and CRE3 KO cells showed no adverse short-term effects mediated by CRISPR/Cas9 editing, as they expanded and maintained their ability to regenerate all four subpopulations (Fig. 7C and Supplementary Fig. S8A–J). In contrast, KO of either CRE6 or of the entire region from CRE3 to CRE6 in the LSC fraction significantly diminished in vitro growth potential, leading to a overall reduction of the number of cells (Fig. 7C), as well as reduction of the four fractions generated by the initially edited CD34+CD38− cells (Supplementary Fig. S8A–J). We additionally performed in vivo functional assays by injecting control and KO cells in NSG-SGM3 mice. All control mice were engrafted 21 weeks after cell injection whereas none of the KO CRE3 or CRE3-6 mice were engrafted (Fig. 7D–F). Taken together, these results establish that KO of CRE3 or KO of the CRE3 to CRE6 region (CRE3-6) caused significant reduction in repopulation potential of the transplanted cells (Fig. 7E, F). Since none of the CRE3 nor CRE3-6 mice were engrafted, this formally established that the KO eradicated all serially transplantable LSC. Overall, these data report the discovery of a new class of stemness regulators within specific non-coding regions of the genome; a result of great value to the stem cell and cancer stem cell community, that exemplifies the utility of OCI-AML22.

**Table 5:** Size of the expected amplicons and additional information for CRISPR editing methods.

Portion to KO	gRNA pairs used		Guides sequence	Expected fragment size if portion KO (bp)	Expected fragment size if portion is NOT KO (bp)	F primer sequence	R primer sequence
CRE3-1	1	3’ guide	TCAAATCATGACACCCTGAA	659	1432	GAAAGTCAGGGCCAGTTGAC	AAGGTTGTCGGGATAAGCGG
		5’ guide	CTGGGCAAGGACCATTCCCA				
CRE3-2	2	3’ guide	TCAAATCATGACACCCTGAA	836	1653	GAAAGTCAGGGCCAGTTGAC	TCTTGGATCTCCAGCCTGTC
		5’ guide	TGTCAGATACAAATCACACC				
CRE3-3	3	3’ guide	TCAAATCATGACACCCTGAA	608	1690	GAAAGTCAGGGCCAGTTGAC	GGAGAACTGCAAGGTTCAGGA
		5’ guide	TAAGCGGTGAAACATTACCA				
CRE6-1	4	3’ guide	ACATCAATTATATTAAACGT	608	1773	CGTCACGTTCTATGAGAGTCC	ACTCCTAGCGACCCATCACT
		5’ guide	caagtagctaggactacagg				
CRE6-2	5	3’ guide	TTCCTTTAGCaaaaaaaaCG	642	1677	AGTCCAGGCATCTGAGGTTC	ACTCCTAGCGACCCATCACT
		5’ guide	caagtagctaggactacagg				
CRE 3 to 6	6	3’ guide	TCAAATCATGACACCCTGAA	637	Portion 1^a^: 1432	GAAAGTCAGGGCCAGTTGAC	AAGGTTGTCGGGATAAGCGG
		5’ guide	caagtagctaggactacagg		Portion 2^a^: 1773	CGTCACGTTCTATGAGAGTCC	ACTCCTAGCGACCCATCACT
					For fragment cut^a^	GAAAGTCAGGGCCAGTTGAC	ACTCCTAGCGACCCATCACT
OR2W5	7	3’ guide	GACAACCAGGAGGACGCACT	500	700	5′-TCGGCCTGGACTGGAGAAAA-3′	5′-GAGACCACTGTGAGGTGAGA-3′
		5’ guide	CTCCCGGTGTGGACGTCGCA				

^a^Given that the region from CRE3 to CRE6 was too long to amplify with classic PCR, only a portion of the region was sequenced using the indicated primers, starting from one side (primers F and R1) or the other side (portion 1) or the other side (portion 2), or the full region in the case same has been KO (for fragment cut), leading to the following expected fragments sizes.

## Discussion

Here, we address the long-standing need for a tool to overcome the challenges of identifying, extracting, and obtaining high numbers of LSC from primary AML samples to enable functional, genetic/epigenetic, biochemical and metabolic studies. OCI-AML22 is the first human AML model that not only faithfully recapitulates the hierarchically organized LSC and non-LSC hallmark states of primary AML samples [8, 9, 20, 21], but also whose LSC fraction can be efficiently identified, isolated and edited using CRISPR editing technology. This sets the stage for mechanistic, functional, and translational studies in the accurate cellular context of the CSC state. Additionally, our study shows how it is now feasible to extend mechanistic studies to uncover stemness regulators lying outside of the coding genome, as demonstrated with functional validation of CRE6 within the LSC+CORE. The OCI-AML22 model shows broad clinical relevance, not just to the relapse sample from which it was derived. The stem cell properties present in the OCI-AML22 LSC population are highly reflective of the stemness properties that are common and highly prognostic from across many highly diverse independent cohorts of primary AML samples [9, 17, 40]. We foresee that the OCI-AML22 model, as well as the extensive dataset generated on the functionally characterized LSC and non-LSC fractions, will be extensively used to interrogate and functionally validate features driving stemness.

CRISPR editing strategies have opened new avenues beyond gene interrogation including editing non-coding elements, which are increasingly being recognized as being regulators of stemness and/or cancer [41]. Non-coding regions are known to harbor cell type specific determinants such as transposable elements or cis-regulatory elements [42]. The importance of these regions in stem cell driven cancers including AML, has been postulated via computational exploration of primary AML sample landscapes [43]. However, these studies did not focus on the rare stem cell population that drives the disease nor did they provide functional validation in primary LSC. Functional validation of non-coding elements has been performed via CRISPR editing but only on AML cell lines [44] that can be easily expanded and genetically modified. However, contrary to the OCI-AML22 model, none of these cell lines reflects an LSC-driven hierarchical structure that mimics primary AML samples. More concerning, after decades in culture, their epigenetic landscape has lost the epigenetic architecture of primary AML samples, limiting the clinical transferability of functional validation performed on these models. Developing and implementing CRISPR/Cas9 editing strategies has not yet been reported in primary AML samples, let alone in rare human LSC, thus the functional proof of the role of CRE in a primary human LSC context with the precision of CRISPR-mediated KO was still missing. Our study provides strong evidence for the functional relevance of non-coding regions that have been predicted through machine learning analysis of matched primary LSC and non-LSC fractions as determinants of stemness. We foresee that leveraging the power of CRISPR with the ability of OCI-AML22 to model stemness determinants that can be extracted from primary AML samples will allow functional interrogations of many more aspects of human LSC features without being limited to the coding genome.

## Supplementary information

Supplementary Figures

Supplementary Figures Legends

Supplementary methods

## Data Availability

Raw data are deposited in the EGA under the series accession number EGAD00001009271 that includes RNA-Seq: EGAS00001006512, ATAC-Seq: EGAS00001006511, WGS: EGAS00001006513. Due to privacy reason, raw data are not available publicly but will be available from the corresponding author on reasonable request. Processed files for RNA-Seq, ATAC-Seq and WGS are deposited under the NCBI’s Gene Expression Omnibus, accessible through GEO series accession GSE211596.
