# Supplementary material for: A primary hierarchically organized patient-derived model enables in depth interrogation of stemness driven by the coding and non-coding genome
Source: Leukemia. 2022 Sep 21;36(11):2690–704. doi: 10.1038/s41375-022-01697-9 (PMC9613464; doi:10.1038/s41375-022-01697-9)
Supplement: Supplementary file 1 — Supplementary Figures [file 41375_2022_1697_MOESM1_ESM.pdf]

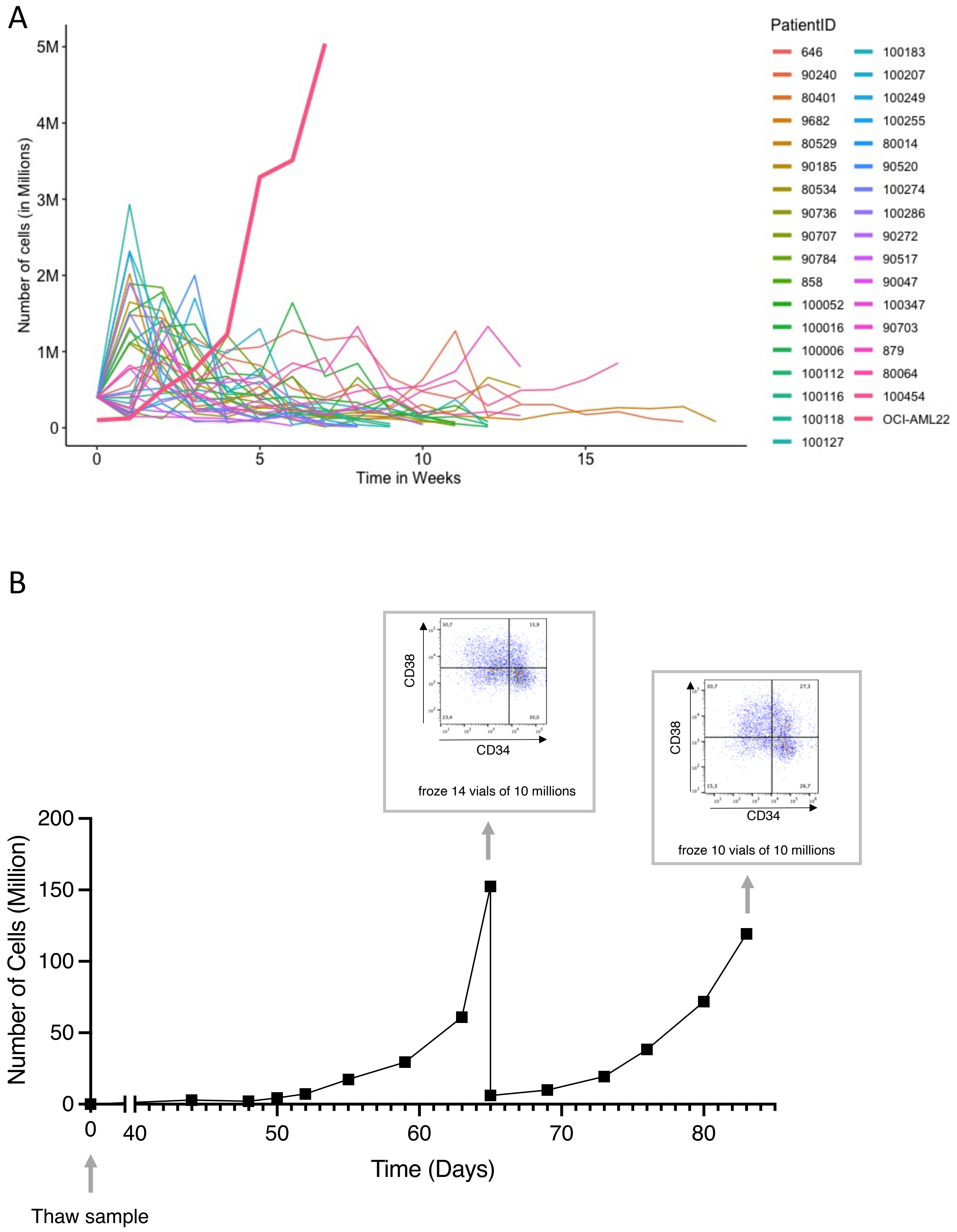

Supplementary Figure S1

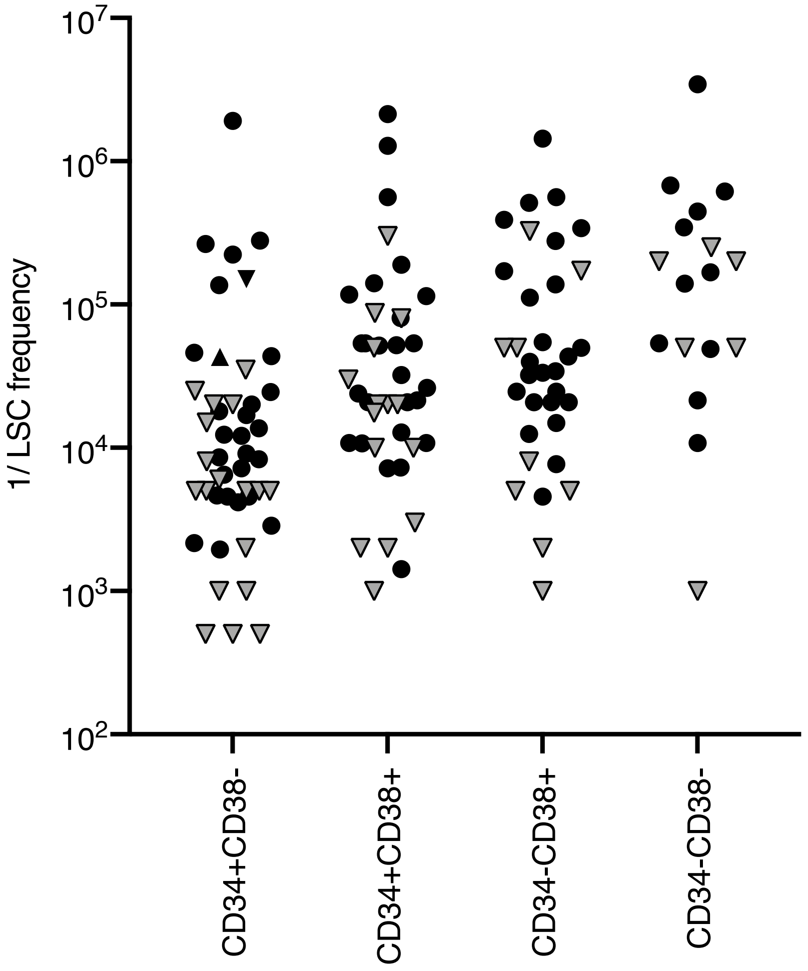

Supplementary Figure S2

A

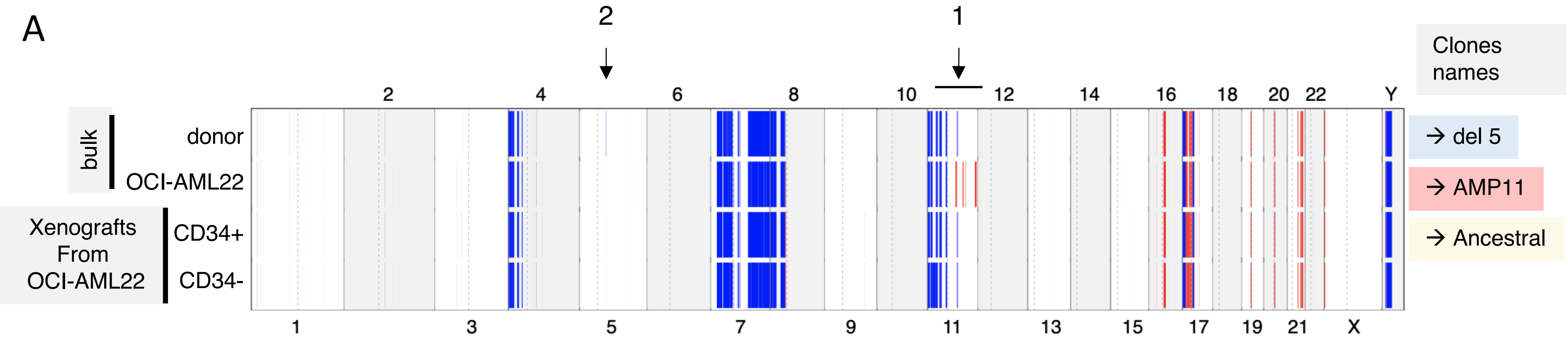

Supplementary Figure S3

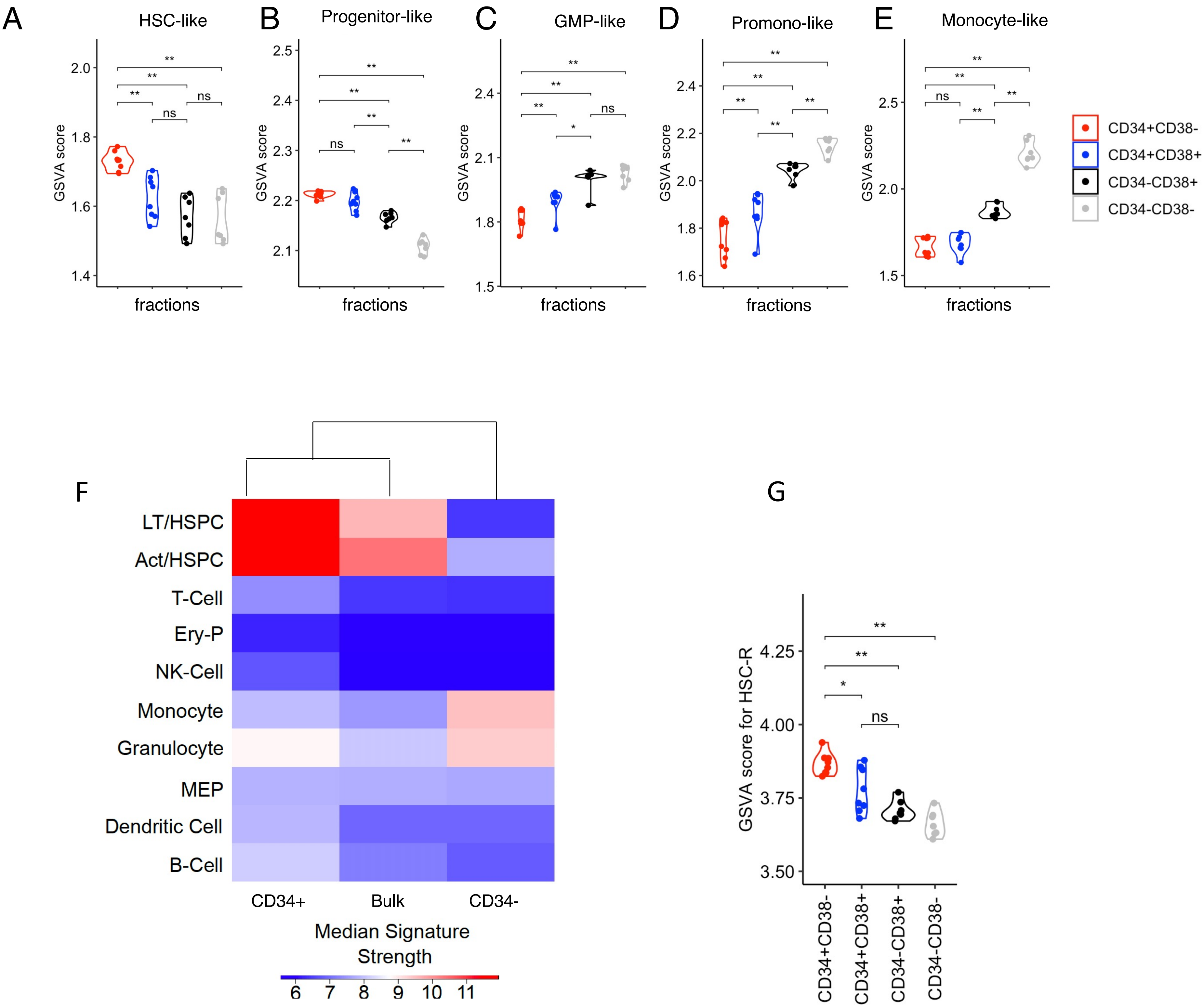

Supplementary Figure S4

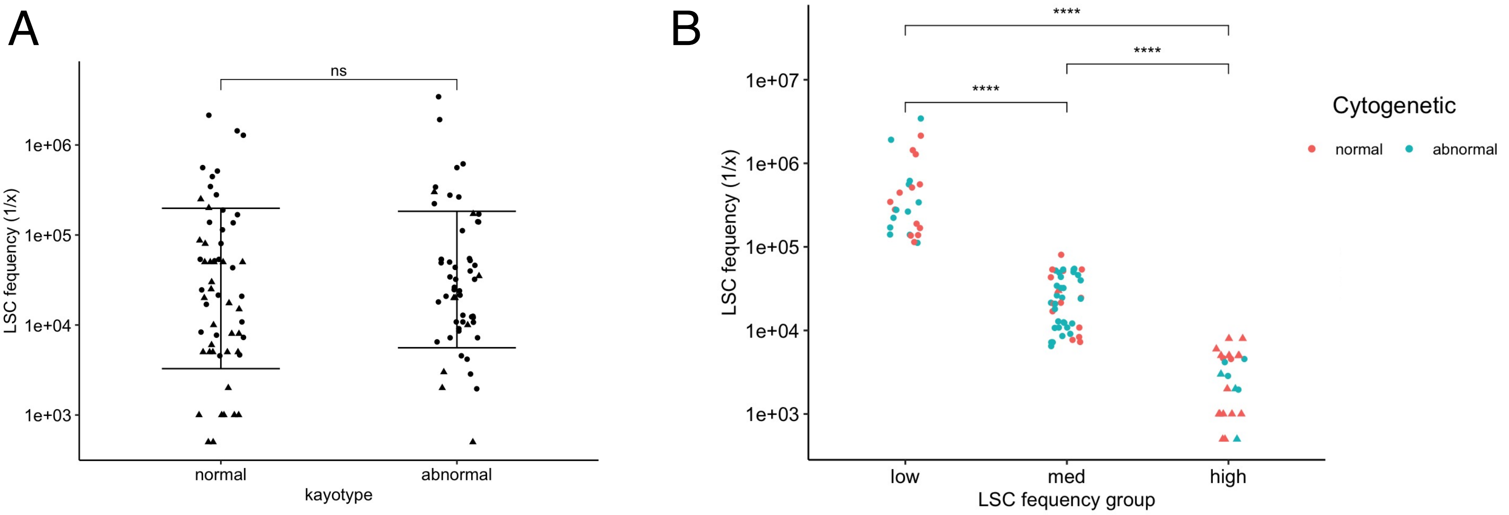

Supplementary Figure S5

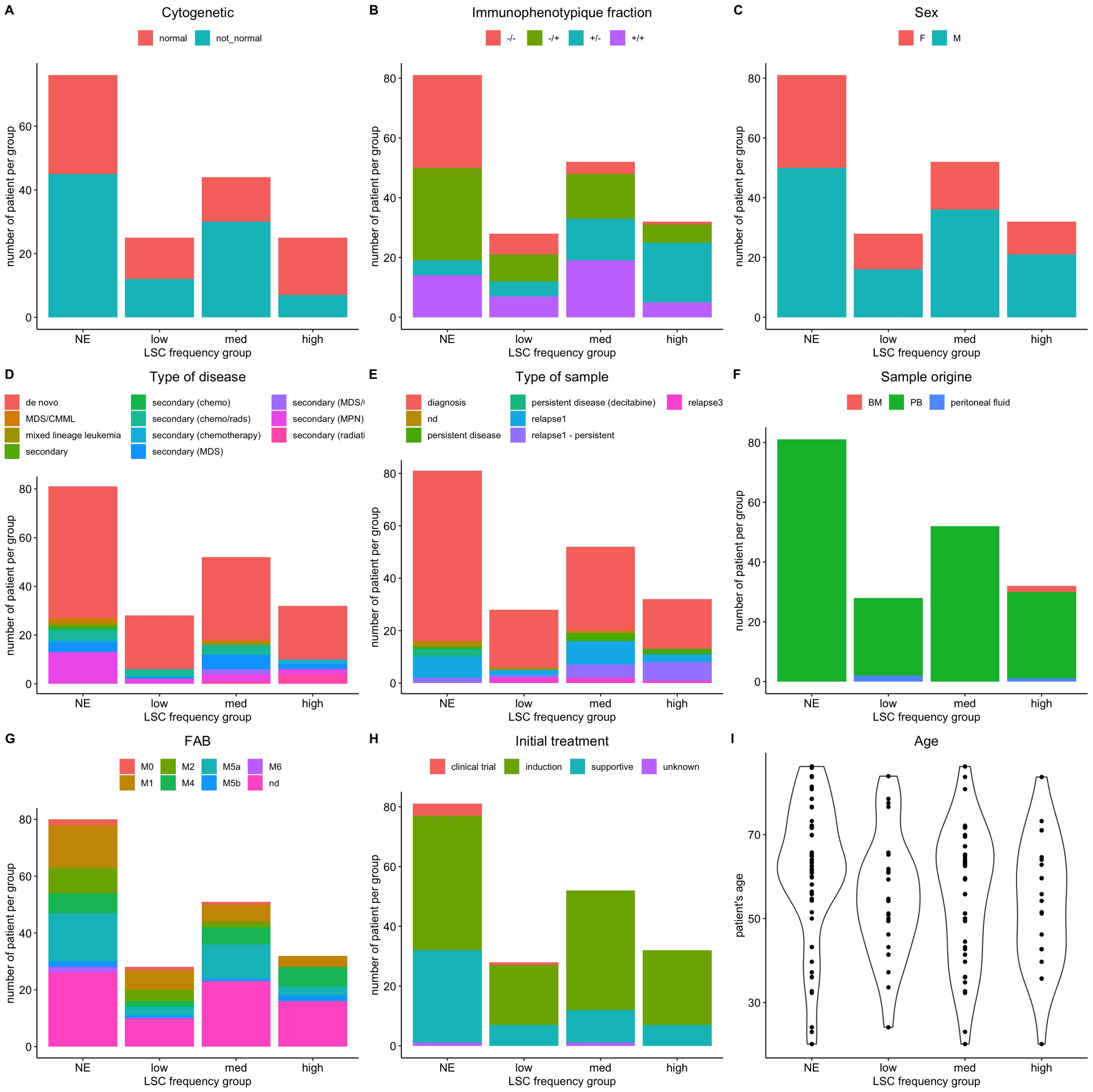

Supplementary Figure S6

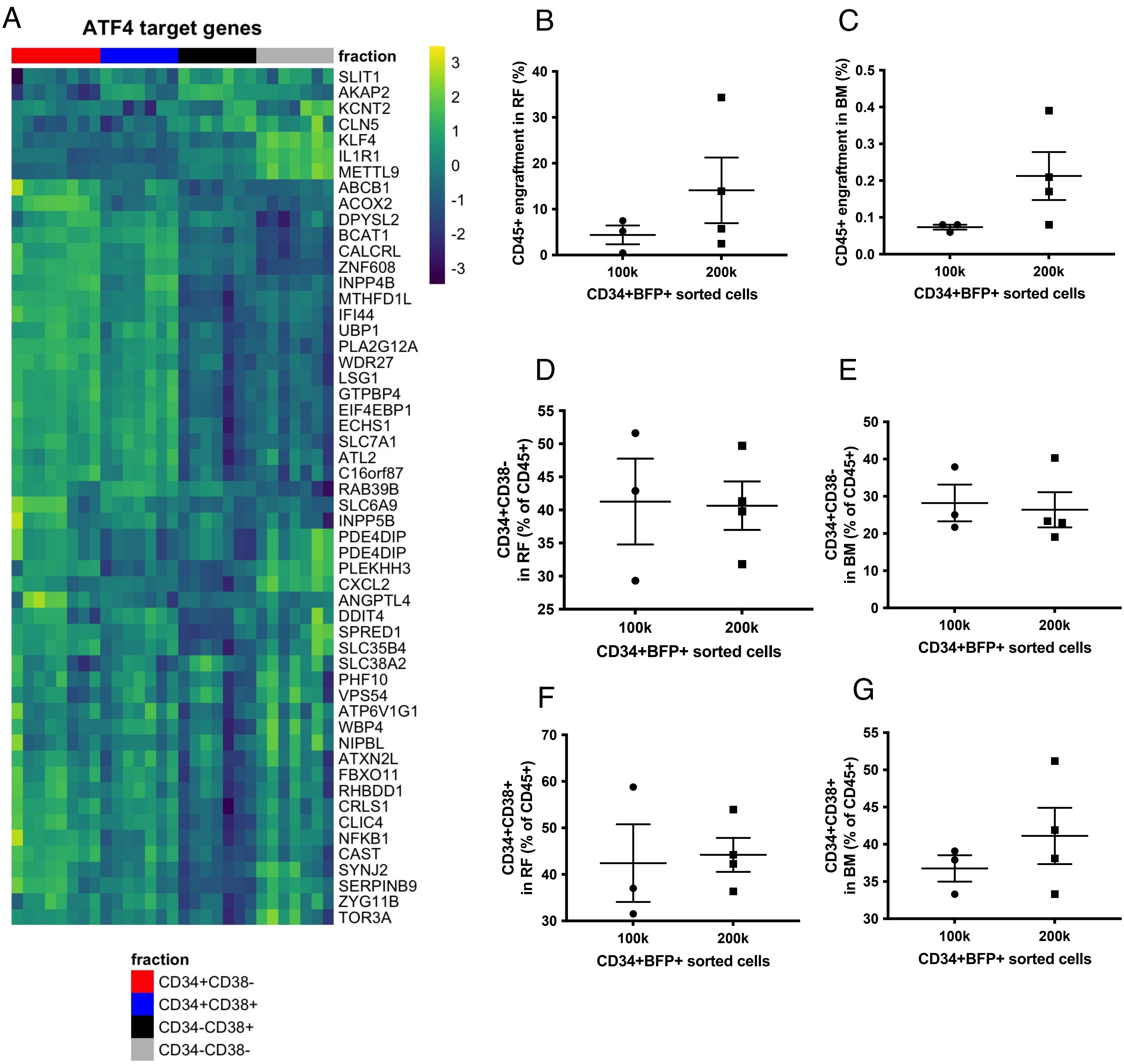

Supplementary Figure S7

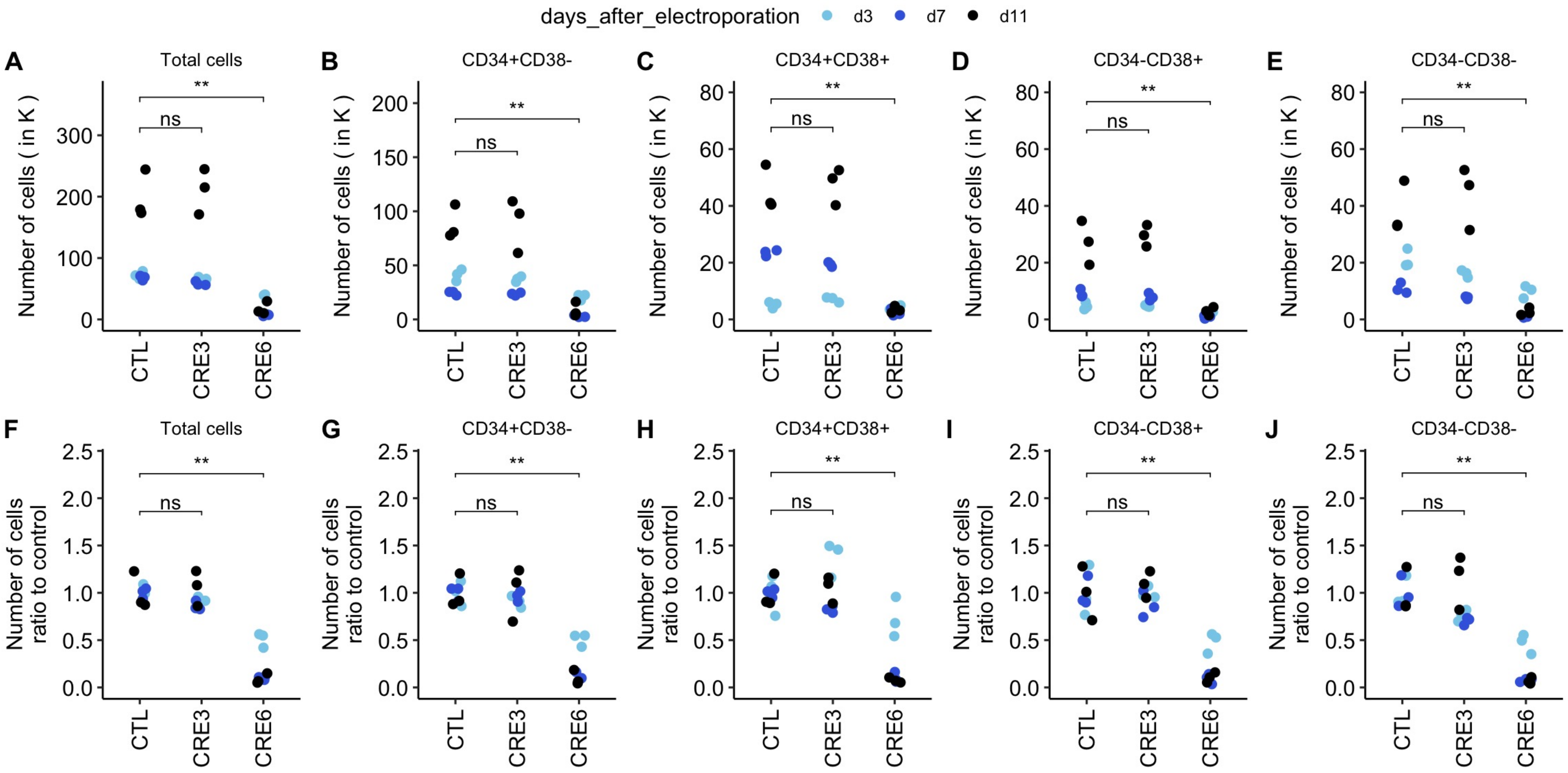

Supplementary Figure S8
