# Supplementary material for: A primary hierarchically organized patient-derived model enables in depth interrogation of stemness driven by the coding and non-coding genome
Source: Leukemia. 2022 Sep 21;36(11):2690–704. doi: 10.1038/s41375-022-01697-9 (PMC9613464; doi:10.1038/s41375-022-01697-9)
Supplement: Supplementary file 2 — Supplementary Figures Legends [file 41375_2022_1697_MOESM2_ESM.docx]

Supplementary Figure legends

**Supplementary Figure 1:** **A**, Primary AML samples unsorted were passed every week using trypan blue dye method. The cell number is indicated on the growth curve for each AML sample. Cells were discarded when the number of cells became lower than the original number of plated cells. The CD34+CD38- OCI-AML22 proliferation curve is indicated in bold pink as a comparison. Cells were cultured as detailed in the methods. **B.** Bulk OCI-AML22 cells were expanded over time as indicated in the methods. Each time cells were frozen the number of cells frozen and the FACS profile is represented.

**Supplementary Figure 2:** **A.** Primary AML samples were sorted based on CD34 and CD38 and injected in NSG mice at various cell doses. 12 weeks after injection, LSC frequency was estimated for each AML fraction (black dot exact LSC frequency, grey triangle upper estimated LSC frequency).

**Supplementary Figure S3: A**. Top line represents the bulk donor sample. the second line represents the bulk OCI-AML22 cultured. Xenografts (CD34+ or CD34- sorted fractions) have been generated 12 weeks after the injection of the cultured OCI-AML22 CD34+CD38- fraction. Copy number losses are defined as CN less than 1.5 (shown in blue) while gains have CN greater that 2.5 (shown in red).

**Supplementary Figure S4**: **A-E**, GSVA score calculated for the *van Galen* signatures, Van Galen, Cell, 2019 : the HSC-like (A), the Progenitor-like (B), the GMP-like (C), the promonocyte-like (D) or the monocyte-like (E) signatures across OCI-AML-22 fractions obtained at different time point as described in Figure 3A. **F,** OCI-AML22 was expanded in culture for about 4 months, before viable CD34+, CD34- fractions or the bulk were sorted and subjected to ATAC-Seq. Signatures previously generated from functionally defined populations of normal hematopoietic cells from the Takayama et al, Cell Stem Cell study, were then applied and signature strength for each of these signatures was calculated. Median z-scores for each signature were used for the heatmap representation. **G**, GSVA scores of genes that make up the HSC-R signature from Eppert, Nature med, 2011 was calculated for each OCI-AML22 sorted fractions.

**Supplementary Figure S5**: **A.** The LSC frequency is represented for each fraction used for either the normal cytogenetic group or the abnormal cytogenetic group. round shape is the exact LSC frequency value, triangle is the upper estimated value since the LSC frequency was not achieved with the dilution done. **B.** The LSC frequency is represented for low, medium (med) and high LSC frequencies groups. round shape is the exact LSC frequency value, triangle is the upper estimated value since the LSC frequency was not achieved with the dilution done. Fractions obtained from normal cytogenetic AML patients are colored in red, fractions obtained from abnormal cytogenetic AML patients are colored in blue.

**Supplementary Figure S6**: **A-I.** Cytogenetic(A), Immunophenotypic fraction (B), Sex (C), type of disease (D), type of sample (E), material of origin (F), FAB classification (G), initial treatment (H) or age (I) of patients that are contributing to each LSC frequency groups used for Figure 3J and 3K

**Supplementary Figure S7**: **A.** Supervised heatmap clustering of ATF4 target genes expression across OCI-AML22 fractions as previously described in Figure 3A. **B-C.** The CD34+CD38- OCI-AML22 fraction was sorted, then transduced with the ATF4 reporter the day after, sorted for BFP+CD34+ cells and injected in mice at 2 different cell doses : 100k and 200k per mice. Engraftment level is displayed for the injected (RF) (B) and non injected (BM) bones (C). **D-E.** The CD34+CD38- OCI-AML22 fraction was sorted, then transduced with the ATF4 reporter the day after, sorted for BFP+CD34+ cells and injected in mice at 2 different cell doses: 100k and 200k per mice. The percentage of CD34+CD38- is displayed for the injected (RF) (D) and non-injected (BM) bones (E). **F-G**. The CD34+CD38- OCI-AML22 fraction was sorted, then transduced with the ATF4 reporter the day after, sorted for BFP+CD34+ cells and injected in mice at 2 different cell doses: 100k and 200k per mice. The percentage of CD34+CD38+ is displayed for the injected (RF) (F) and non injected (BM) bones (G).

**Supplementary Figure S8**: **The OCI-AML22 LSC fraction can be CRISPR edited: A-J.** absolute number of cells 3, 7 or 11 days after electroporation of 100k CD34+CD38– OCIAML22 cells, showing the total number of cells (A), Number of CD34+CD38– cells (B), Number of CD34+CD38+ cells (C), Number of CD34–CD38+ cells(D), Number of CD34–CD38– cells(E). Ratios to the control are indicated below each graph (F-J**).**
