# Supplementary material for: A primary hierarchically organized patient-derived model enables in depth interrogation of stemness driven by the coding and non-coding genome
Source: Leukemia. 2022 Sep 21;36(11):2690–704. doi: 10.1038/s41375-022-01697-9 (PMC9613464; doi:10.1038/s41375-022-01697-9)
Supplement: Supplementary file 3 — Supplementary methods [file 41375_2022_1697_MOESM3_ESM.docx]

Supplementary material

**Primary samples**

Primary AML samples were collected with written informed consent according to the procedures approved by the University Health Network (UHN) Research Ethics Board (REB 01-0573-C). 400 000 cells were initially plated at 2 millions cells per ml for each tested sample. Cells were maintained in culture for the indicated time in the following medium: X-VIVO 10 (Lonza, BE04-380Q) supplemented with 20% BIT 9500 Serum Substitute (StemCell technologies, 09500), SCF (50 ng/ml) (Miltenyi Biotech, 130-096-696), IL3 (25 ng/ml)( Miltenyi Biotech, 130-095-069), TPO (50 ng/ml; Peprotech, 300-18), FLT3L (50 ng/ml; Peprotech, 300-19 B), IL6 (25 ng/ml; Miltenyi Biotech, 130-093-934), G-CSF (25 ng/ml; Miltenyi Biotech, 130-093-861).

**Clinical characteristics of the patient from which OCI-AML22 originated**

Male, 58 old, diagnosed with acute myeloid leukemia with myelodysplasia related changes of unclassified FAB carrying TP53 mutation (variant C743G>A), VAF 0.939. Karyotype of the patient at diagnosis was 46,XY,del(3)(p21),+del(3)(q21),+del(6)(q21),del(7)(q22q32),-11,-14,-17,+mar[9].

**Disease history of the donor that generated OCI-AML22 and OCI-AML22 establishment**

The donor that generated the OCI-AML22 model had a primary refractory response to the classical 7+3 based induction chemotherapy combining daunorubicin and cytarabine. It was followed by a FLAG-IDA re-induction. After an initial clinical remission, the patient relapsed 55 days later. He then received NOVE HiDAC re-induction therapy, followed by haploidentical hematopoietic stem cell transplantation, which led to a second remission. He then relapsed 78 days after transplantation. Following this second relapse, the patient started a leukoreduction protocol based on azacytidine and hydroxyurea. At that point, a peripheral blood sample was collected and blasts were isolated to generate the OCI-AML22 sample. Later, the patient was treated with Myelotarg, but complete remission was not achieved. Overall survival was 480 days.

The peripheral blood from the donor was subjected to ficoll separation. At that time, the percentage of blasts was 89%. Freshly obtained cells were then expanded in culture in the following medium, with final concentrations as indicated : X-VIVO 10 (Lonza, BE04-380Q) supplemented with 20% BIT 9500 Serum Substitute (StemCell technologies, 09500), 1x Glutamax Supplement (Thermo Fisher Scientific, 35050061), Primocin 0.1 mg/ml (invivogen, ant-pm-1), SCF (200 ng/ml;Miltenyi Biotech, 130-096-696), IL3 (20 ng/ml;Miltenyi Biotech, 130-095-069), TPO (20 ng/ml; Peprotech, 300-18), FLT3L (40 ng/ml; Peprotech, 300-19 B), IL6 (10 ng/ml; Miltenyi Biotech, 130-093-934), G-CSF (10 ng/ml; Miltenyi Biotech, 130-093-861). Cells were maintained at a density of 0.8x10^6^ cells/ml and passaged twice a week, in a 96 well flat bottom plate surrounded by PBS on the outside wells. A full medium change was done once a week. The CD34+CD38- fraction or CD34+ fraction was regularly sorted to serially expand the cells.

**Cytogenetic of the donor that generated the OCI-AML22 model**

The donor cells display a complexe cytogenetic that is characterized by:

- A series of deletions on chromosomes 4, 7, 8, 11.
- A series of amplifications on chromosome 11.
- Copy number alterations on chromosome 17. Of note deletion of chromosome 17 is also consistent with the TP53 mutation LOH observed on the diagnosis sample.
- A series of amplifications of chromosome 21.
- Partial chromosome Y deletion

**Xenotranplantation**

All animals used for this study were treated in accordance with institutional guidelines and ethical regulations (approved in AUP number 1117, UHN). Female and male NSG mice (NOD.Cg PrkdcscidIl2rgtm1Wjl/SzJ; The Jackson Laboratory) or male NSG-SGM3 mice, as indicated in figure legends were irradiated with 250 rads the day before intrafemoral injection. Animals were 10-12 weeks old before injection. Bulk cells expanded in vitro for an average of 4 months were used throughout this study unless specified otherwise and are considered the OCI-AML22 model. Fractions were obtained from expanded cells in vitro after an average of 4 months (unless specified otherwise), then sorted according to their CD34 and CD38 expression, using AnnexinV-FITC (556419, BD) and 7-AAD (559619, BD) to exclude apoptotic and dead cells. The number of injected cells per experiment is indicated in the figure legends. For lentiviral transduction experiment, cells were transduced with the ATF4 reporter as described in [^37^](https://paperpile.com/c/Dujh77/19ctq) in their standard culture medium overnight at a density of 0.8x10^6^ cells/ml, washed and injected the day after. Mice were euthanized and the injected femur (right femur /RF) and the non-injected left femur as a surrogate for engraftment in the bone marrow (BM) were flushed separately in MEMalpha with 10% FBS. Engraftment level was assessed by flow cytometry on a FACSCelesta instrument (BD) with the following antibodies: mCD45-FITC (553080, BD) or hCD45-FITC (561865, BD, clone HI30) combined with hCD45-APC-Cy7 (624072, BD, clone 2D1), hCD34-PE (348057, BD), hCD38-PE/Dazzle (303538, BD) and 7-AAD (559619, BD). 5 mouse per conditions where used for each experiment, injected at the same time point. For secondary engraftment, human cells collected from NSG engrafted mice were sorted using 7-AAD (559619, BD), AnnexinV-FITC (556419, BD), hCD45-APC-Cy7 (624072, BD), then injected into NSG-SGM3 mice (n=5 mice per group) at the indicated cell dose per mice and sacrificed at 8 weeks after. The engraftment level was determined as for the primary engraftment assay. Mice were randomly attributed to each group before injection. No blinding was done.

**RNA-seq**

Primary cells were expanded generating the OCI-AML22 model and collected at different time points to sort the resulting subfractions based on CD34 and CD38 cell surface expression as indicated in figure legends. Total RNA was extracted using the mirVana™miRNA Isolation Kit, with phenol (Invitrogen) as recommended. Samples that passed quality control according to integrity (RIN>8) and concentration as verified on a Bioanalyzer pico chip (Agilent Technologies) were subjected to further processing by the Center for Applied Genomics, Sick Kids Hospital. The SMART-Seq v4 Kit (SSv4) (Takera) followed by [Illumina Nextera XT library prep](https://www.illumina.com/products/by-type/sequencing-kits/library-prep-kits/nextera-xt-dna.html) was used as recommended by the manufacturer. Briefly, the same amount of RNA was used for library preparation. After cDNA conversion, cDNA was run on a bioanalyzer. 1ng of cDNA was used for the Nextera library preparation, then subjected to QC on a bioanalyzer and qPCR for the final library concentration. For sequencing, all libraries were pooled at equimolar amounts, followed the illumina protocol to dilute the pool to the appropriate concentration for sequencing. All samples were sequenced in parallel to avoid batch effects, on the NovaSeq 6000, S1 flow cell, PE100bp. Approximately 43 million paired reads per sample were generated.

**RNA-seq analysis**

Sequencing was aligned using STAR 2.5.2b [^45^](https://paperpile.com/c/Dujh77/geBcb) against GRCh38 and transcript sequences downloaded from Ensembl build 90. Default parameters were used except for the following: “--chimSegmentMin 12 --chimJunctionOverhangMin 12 --alignSJDBoverhangMin 10 --alignMatesGapMax 100000 --alignIntronMax 100000 --chimSegmentReadGapMax parameter 3 --alignSJstitchMismatchNmax 5 -1 5 5”. Counts were obtained using HTSeq v0.7.2. Variance stabilized normalized counts were generated. The variance stabilized normalized counts was generated by DESeq2 v1.22.2. Gene set enrichment was performed using GSEA PreRanked v3.0 ([http://www.broad.mit.edu/gsea/](https://www.gsea-msigdb.org/gsea/index.jsp)) against the indicated gene sets as specified in figure legend (settings : method “ssgea, kcdf : “Gaussian”). The UMAP package in R [^46^](https://paperpile.com/c/Dujh77/9f8We) was used (parameters: number of neighbors = 5, min dist = 0.01) to reduce the dimensionality of (Figure 7G-I). Clusters were identified using the k means method. PCA has been generated using the plotPCA function from DESeq2 v1.22.2 package and the top 1000 variable genes. Graphic has been made using ggplot2.

**Generation of references stem cell signatures used in Figure 4**

The normal karyotype LSC+ signature has been generated by taking the average gene expression of each of the 104 genes part of the LSC104 signature, using the 60 LSC+ fractions obtained from patients with normal karyotype and obtained from [^9^](https://paperpile.com/c/Dujh77/AoQYf). The abnormal karyotype LSC+ signature has been generated by taking the average gene expression of each of the 104 genes part of the LSC104 signature, using the 55 LSC+ fractions obtained from patients that did not carry a normal karyotype and obtained from [^9^](https://paperpile.com/c/Dujh77/AoQYf). The LSC frequency High signature was generated by taking the average gene expression of each of the 104 genes part of the LSC104 signature, using the 32 LSC+ fractions that presented the highest LSC frequency within our entire cohort of 166 functionally assessed fractions, obtained from previous work [^9^](https://paperpile.com/c/Dujh77/AoQYf). The LSC frequency Med signature was generated by taking the average gene expression of each of the 104 genes part of the LSC104 signature, using the 52 LSC+ fractions that presented a medium LSC frequency within our entire cohort of 166 functionally assessed fractions, obtained from previous work [^9^](https://paperpile.com/c/Dujh77/AoQYf). The LSC frequency Low signature was generated by taking the average gene expression of each of the 104 genes part of the LSC104 signature, using the 28 LSC+ fractions that presented a low LSC frequency within our entire cohort of 166 functionally assessed fractions, obtained from previous work [^9^](https://paperpile.com/c/Dujh77/AoQYf). LSC frequencies for each of these categories are detailed in Supplementary Figure S5B, and are representative of a large panel of AML samples as presented in Supplementary Figure S6.

**ATAC-Seq library preparation**

Library preparation for ATAC-Seq was performed by the the “Princess Margaret Genomics Centre, Toronto, Canada ([www.pmgenomics.ca](http://www.pmgenomics.ca/)) on 100,000 sorted CD34+CD38- OCI-AML22 cells that have been cultured for about 4 months (n=1 per population), with Nextera DNA Sample Preparation kit (FC-121-1030, Illumina), according to previously reported protocol [^47^](https://paperpile.com/c/Dujh77/yTTyc). Libraries for ATAC were sequenced on the Novaseq 6000 System (Illumina) to generate paired-end 50-bp reads.

**ATAC-Seq analysis**

Unaligned reads were mapped against the hg19 human reference genome using BWA [^48^](https://paperpile.com/c/Dujh77/YKrq1) with default parameters. All duplicate reads, and reads mapped to mitochondria, chrY, an ENCODE blacklisted region or an unspecified contig were removed [^49^](https://paperpile.com/c/Dujh77/8trdD). MACS 2.0.10 [^50^](https://paperpile.com/c/Dujh77/kn0K7) callpeak was used to call peaks from aligned reads, and called peaks from MACS2 were used as input for CREAM (ref). MACS2 bdgcmp was used to obtain the fold-enrichment over background and subsequently visualized in IGV following conversion to TDF format with IGVtools.

**Lentiviral production of ATF4 reporter**

The ATF4 reporter was used as previously reported [^20^](https://paperpile.com/c/Dujh77/jPigb). Pseudotyped lentiviral particles were produced and titers calculated as previously described [^37^](https://paperpile.com/c/Dujh77/19ctq). Lentivirus were concentrated 100x by ultracentrifugation, resuspended in X-VIVO 10 supplemented with 1% BSA and stored at – 80C until use. Cells were transduced overnight and washed the day after with a fresh medium before their injection in mice. Cells were transduced with the same lentivirus batch.

**CRISPR editing of the OCI-AML22 LSC fraction**

The protocol was adapted from[^51^](https://paperpile.com/c/Dujh77/zMCAs) for CRISPR editing of LSCs. For each region to knock out, pairs of guides Alt-R CRISPR/Cas9 crRNA synthetized from IDT were used to knock out either: the control OR2W5 olfactory gene, the CRE3 element (chr 9, 2022600-2022800, hg19), the CRE6 element (chr 9 2026800-2027000, hg19), or the entire region from CRE3 to CRE6 (chr 9, 2022600-2027000, hg19).

Guides for each of the pairs, indicated in the table, where annealed with tracrRNA (IDT, 1072533) in a ratio 1crRNA, 1crRNAg2, 2 tracrRNA, at 95 °C for 5 min, then cooled down to room temperature. Then 1.2uL of the resulting functional gRNA duplex was combined with 1.7uL cas9 protein (IDT, 1081059) and 2.1uL PBS and incubated for 15 min at room temperature. 1uL of electroporation enhancer (IDT, 1075916) was subsequently added in each reaction to generate the complex mix. In parallel, cultured OCI-AML22 cells, pre-sorted the day before for its CD34+CD38- fraction using the [MoFlo XDP U/VBR](https://ca.ilab.agilent.com/schedules/357581#/schedule) Beckman Coulter sorter, and incubated overnight in their own medium, were washed with room temperature PBS using a benchtop centrifuge at 3rpm for 2 minutes. 100 000 cells per reaction were then resuspended in 20uL of P3 solution (Lonza, V4XP-3032) and mixed by pipetting with the full gRNA-Cas9 complex mix previously assembled then added in the electroporation chamber well (Lonza, V4XP3032). Cells were electroporated with the program EO-100 using the Lonza Nucleofector. Then, 180 μl of pre-warmed OCI-AML22 media was added. Cells were transferred in a 96 plate well surrounded by PBS to maintain humidity, then left overnight in the incubator before replacing 100uL of old medium with fresh OCI-AML22 media. After each CRISPR/Cas9 RNP electroporation, a small subset of cells was cultured in the OCI-AML22 media for about a week to obtain enough cells for DNA analysis. Genomic DNA was isolated for each condition, using the Agencourt GenFind V2 (Beckman Coulter, A41499). The CRISPR/Cas9 engineered genomic locus was amplified via PCR as previously described [^51^](https://paperpile.com/c/Dujh77/zMCAs). For each PCR reaction, 23 μl of eluted genomic DNA was mixed with 1 μl of forward and reverse primer (10 μM) and 25 μl of AmpliTaq Gold 360 Master Mix (ThermoFisher, 4398881). The PCR program was: 95 °C for 10 min, followed by 95 °C for 30 s, 56 °C for 30 s and 72 °C for 1 min (40 cycles) and then 72 °C for 7 min. PCR primers used are indicated in the table. The experiment was repeated 3 times.

A portion of the PCR amplicon was run on an agarose gel to verify both PCR specificity and cutting efficiency at deleting a fragment for each pair of guides used, as specified above (see gel, Figure 4I). Size of the expected amplicons for the cut or uncut region is specified in Table 5.

The rest of PCR products were purified using the MinElute PCR Purification Kit (Qiagen, 28004). Sanger sequencing was performed using the same F primers used for PCR amplification. Chromatograms were analyzed using the online tool TIDE (<https://tide.deskgen.com/>)[^52^](https://paperpile.com/c/Dujh77/4OSKQ) to verify that the break site is at the expected location which validates precision of the cut.

**Whole genome sequencing**

Genomic DNA was extracted using the QIAamp DNA Micro kit (Qiagen, ref 56304) following recommendations. Whole Genome Libraries, one hundred nanogram of sorted cell DNA from each sample was fragmented using a Covaris M220 Focused-ultrasonicator. Libraries were then generated using KAPA HyperPrep Kit (Roche, Cat# 07962363001) according to manufacturer’s instruction. The final purified libraries were balanced and pooled and sequenced on an Illumina NovaSeq at paired end 150 cycles.

**Whole genome sequencing analysis**

We used 2 independent pipelines: HMMcopy (<https://bioconductor.org/packages/release/bioc/html/HMMcopy.html>) and celluloid  [^53^](https://paperpile.com/c/Dujh77/2AYth), to detect copy number abnormalities. The whole genome sequencing data was aligned to hg38 using bwa v0.7.12 with default settings. Reads were sorted and duplicates were marked with picard v2.21.4. Copy number calls were made using both HMMcopy v0.1.1 and celluloid v0.11.7 with 1kb windows. Telomeric and centromeric regions were removed from further analysis. We considered anything with a copy number below 1.5 as a loss and greater than 2.5 as a gain. Using bedtools v2.29.2 we identified overlapping regions in pairwise fashion to determine the percentage of overlap between each sample. To determine if subclones were present, we focused on the alterations found on region of chr11 q arm. Using the same segments identified in bulk_1m, we compared the distribution of the binned copy number values between adjacent segments using a wilcox test. This was done in all samples to identify if there was a small change between segments that were not identified by the copy number callers.

**Statistical analysis**

GraphPad Prism or R was used. Unless specified, Mann Whitney test was performed. *<0.05, **p<0.01, ***p<0.001, and variance was checked to be similar between the groups that are compared.

**Data availability statement**

Raw data are deposited in the EGA under the series accession number EGAD00001009271 that includes RNA-Seq: EGAS00001006512, ATAC-Seq: EGAS00001006511, WGS: EGAS00001006513. Due to privacy reason, raw data are not available publicly but will be available from the corresponding author on reasonable request.

Processed files for RNA-Seq, ATAC-Seq and WGS are deposited under the NCBI’s Gene Expression Omnibus, accessible through GEO series accession GSE211596.
